# Orthogonal Supramolecular Assemblies Using Side-Chain Functionalized Helical Poly(isocyanide)s

**DOI:** 10.1021/acs.macromol.2c02224

**Published:** 2023-05-12

**Authors:** Chengyuan Wang, Ru Deng, Marcus Weck

**Affiliations:** Department of Chemistry and Molecular Design Institute, New York University, New York, New York 10003, United States

## Abstract

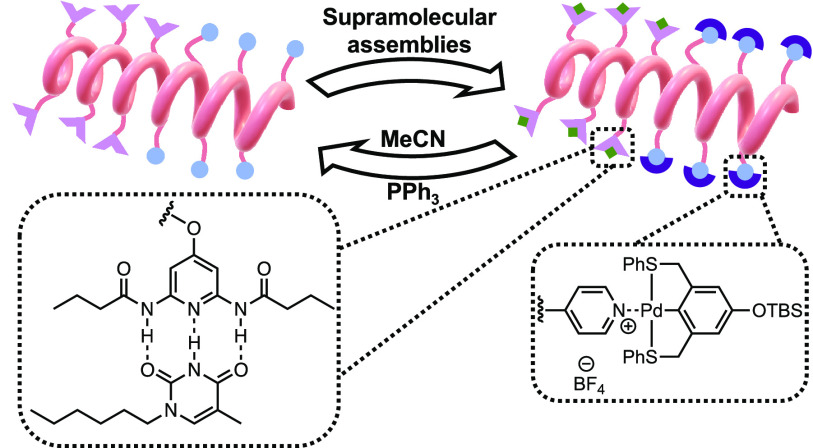

Mimicking the structure of proteins using synthetic polymers requires building blocks with structural similarity and the use of various noncovalent and dynamic covalent interactions. We report the synthesis of helical poly(isocyanide)s bearing diaminopyridine and pyridine side-chains and the multistep functionalization of the polymers’ side-chains using hydrogen bonding and metal coordination. The orthogonality of the hydrogen bonding and metal coordination was proved by varying the sequence of the multistep assembly. The two side-chain functionalizations are reversible through the use of competitive solvents and/or competing ligands. Throughout the assembly and disassembly, the helical conformation of the polymer backbone is sustained as proved by circular dichroism spectroscopy. These results open the possibility to incorporate helical domains into complex polymer architectures and create a helical scaffold for smart materials.

## Introduction

Nature’s delicate design of biopolymers such as proteins and DNA presents chemists with a plethora of targets to study and mimic. With only 22 natural α-amino acids as the basic building blocks, proteins exhibit a rich variety of structures and diverse functions due to their hierarchical 3D structures. The primary sequence of amino acids and the secondary conformation of proteins such as α-helices or β-sheets contribute to the formation of precise tertiary architectures that endow proteins with functions.^1,2^ Peptide domains with specific secondary structures fold into tertiary structures through precise arrangement of orthogonal and/or synergistic noncovalent interactions such as hydrogen-bonding, Coulombic and hydrophobic interactions, and dynamic covalent bonds (e.g., disulfide bond).^3^ The remarkable structure–function relationship of proteins inspired the exploration of synthetic polymers as biomimetic materials on various scales such as monomer sequence, secondary conformations, tertiary structures, nanoscale assemblies, and beyond.^4,5^

In the synthetic realm, chemists have created a large library of polymer backbones beyond the amide- and ester-based polymers that are prevalent in nature, enabling a variety of complex materials.^6^ Compared to natural peptides, synthetic polymers have the advantages of a more diverse set of monomers, often better tolerance to harsh environmental conditions, and a large variety of polymerization methods.^4^ Synthetic polymers, however, usually are not monodisperse, and control over the monomer sequence is limited.

Multiple strategies have been reported to mimic the structures and functions of proteins. One polymer class developed toward this goal is single-chain polymer nanoparticles (SCNPs)^7−9^ which have been utilized to not only develop structural mimetics but also find applications in catalysis^10−14^ and drug delivery.^15,16^ SCNPs have defined tertiary structures in spherical shapes without secondary structure domains such as helical or sheet-like blocks. A notable exception to this is the helical supramolecular assembly of benzene-1,3,5-tricarboxamide,^17,18^ which can form helical domains within a SCNP. While many synthetic polymers exhibit defined secondary structures such as helical poly(isocyanide)s,^19−21^ helical poly(phenyl acetylene)s,^20,22^ and sheet-like poly(*p*-phenylenevinylene),^23−25^ the case of incorporating them into synthetic tertiary architecture is still limited, which is mainly due to a lack of functionalized side-chains that can interact directionally and specifically with other domains. We have reported main-chain block copolymers^26−31^ and miktoarm star polymers^32^ using these secondary structure-based building blocks in combination with molecular recognition units (MRUs) and covalent chain-extension strategies. To move these architectures with rich secondary structures into the realm of tertiary structures, strategies that enable functionalization of side-chains of the building blocks with multiple MRUs that can direct the interactions between multiple domains are key.

**Figure 1:**
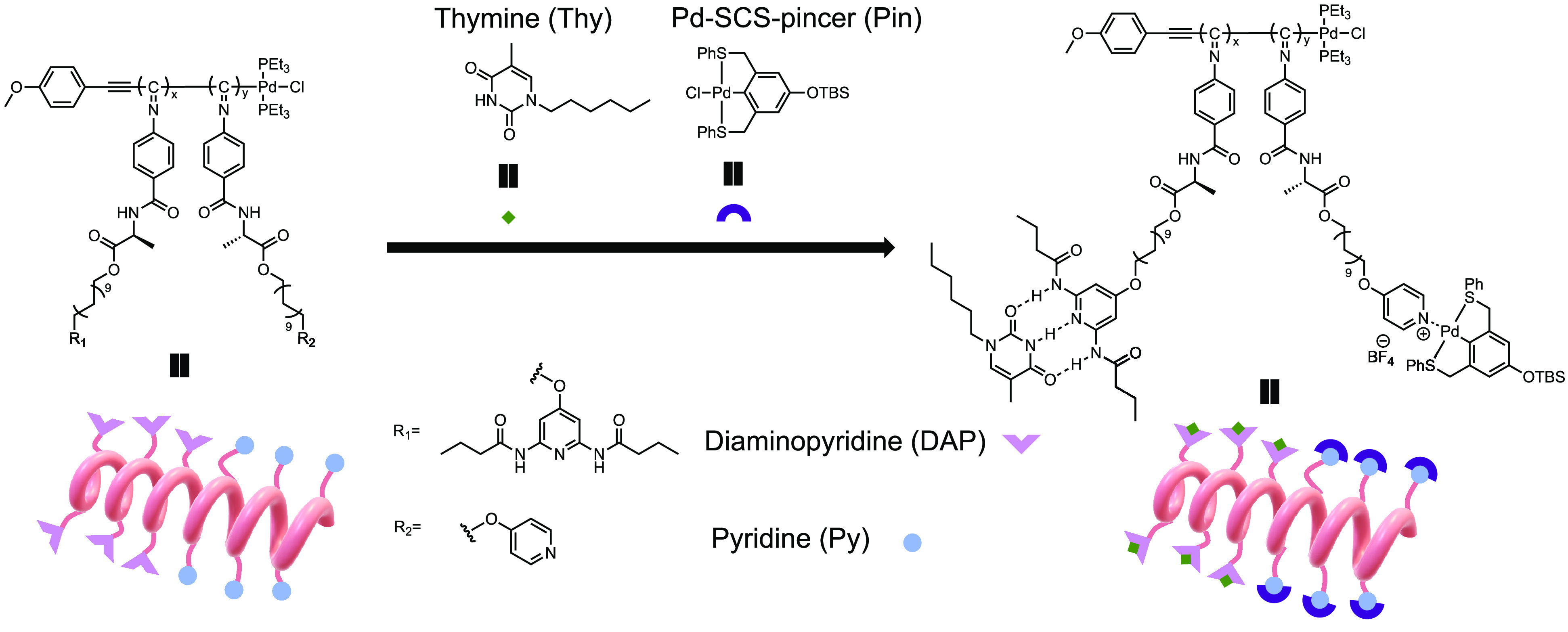
Schematic representation of the orthogonal supramolecular assembly design.

As the most abundant secondary domain element in proteins, the α-helix plays an indispensable role in maintaining the structure and function of proteins. Inspired by the α-helix, chemists have investigated synthetic helical polymers. Among the well-studied synthetic helical polymers,^20^ poly(isocyanide) is a static helical polymer that can be prepared in a controlled manner with a high density of functionalizable side-chains.^19,21^ Helical polymers with supramolecular recognition units that can form complexes with specific complementary moieties on their side-chains are rare.^33−37^ We reported the side-chain functionalization of helical polymers through metal coordination using a poly(isocyanide) random copolymer bearing pyridine side-chains.^38^ As a single MRU might be insufficient to realize directional folding of a multicomponent polymer system to a true tertiary structure, we hypothesize that multiple MRUs need to be incorporated for a controlled folding process. This contribution describes such a system: helical poly(isocyanide)s functionalized with two different types of MRUs which allow for the noncovalent functionalization and assembly based on hydrogen-bonding interactions and metal coordination (Figure 1). Our MRUs are the pyridine (Py)-palladated sulfur–carbon–sulfur pincer (Pin) metal coordination pair and the diaminopyridine (DAP)–thymine (Thy) hydrogen-bonding pair that have been shown by us previously to be orthogonal.^39−41^ The DAP-Thy pair assembles via hydrogen bonds, and the assembly can be disrupted by competing hydrogen bond donors and acceptors. The Py-Pin pair is a MRU pair that requires a silver salt such as AgBF_4_ to trigger the metal coordination. Combining these two motifs, we aimed for a helical polymer scaffold with side-chain tunability and dynamics through supramolecular assembly.

## Experimental Section

### Materials and Instrumentation

All chemicals were purchased from Sigma-Aldrich, TCI, and Oakwood Chemical and used as received unless otherwise indicated. Gel-permeation chromatography (GPC) was done using a Shimadzu pump coupled to a Shimadzu RI detector. Poly(styrene) standards purchased from Agilent Technologies were used for column calibration. Two GPCs with different eluents were used. One GPC ran with a 0.03 M LiCl solution in *N*,*N*-dimethylformamide (DMF) as the eluent at a flow rate of 1 mL/min at 65 °C. A set of Polymer Standards columns (AM GPC gel, 10 μm, precolumn, 500 Å, and linear mixed bed) was used. The second GPC ran with THF as the eluent at a flow rate of 1 mL/min at ambient temperature. A set of Shodex GPC columns (KF-804 and KF-802.5) was used. *M*_w_, *M*_n_, and *Đ* represent respectively the apparent weight-average molecular weight, apparent number-average molecular weight, and dispersity index. ^1^H NMR and ^13^C NMR spectra were recorded at 25 °C on a Bruker AVIII400 MHz, a Bruker AV 500 MHz, or a Bruker AVIII 600 MHz spectrometer. All chemical shifts are reported in parts per million (ppm) with reference to solvent residual peaks. Mass spectra of samples in methanol were acquired with an Agilent 6224 Accurate-Mass TOF/LC/MS spectrometer. Circular dichroism (CD) spectra and UV–vis spectra were obtained at 25 °C on a Jasco J-1500 circular dichroism spectrometer.

### General Polymerization Procedure

**M1** (80 mg, 0.13 mmol) and Pd initiator (2.14 mg, 4.19 μmol) were dissolved in THF (0.63 mL) in a Schlenk flask, and three freeze–pump–thaw circles were applied to degas the reaction. The flask was then transferred to an oil bath, and the reaction mixture was stirred at 55 °C for 24 h. The solvent was removed under reduced pressure. The crude product was dissolved in a minimal amount of THF and was precipitated with methanol. The dissolution and precipitation procedure was repeated three times, and the product was obtained by filtration and dried under vacuum as a brown solid (68 mg, yield 83%).

### Block Copolymerization Procedure

**M1** (95 mg, 0.15 mmol) and the Pd initiator (3.80 mg, 7.46 μmol) were dissolved in THF (0.75 mL) in a Schlenk flask, and three freeze–pump–thaw circles were applied to degas the reaction. The flask was then transferred to an oil bath, and the reaction mixture was stirred at 55 °C for 24 h. After 24 h, **M2** (139 mg, 0.30 mmol) in degassed THF (0.75 mL) was injected into the reaction flask using a syringe. The reaction was heated for an additional 48 h. The solvent was removed under reduced pressure. The crude product was dissolved in a minimal amount of THF and was precipitated with methanol. The dissolution and precipitation procedure was repeated three times, and the product was obtained by filtration and dried under vacuum as a brown solid (196 mg, yield 82%).

### Hydrogen-Bonding Procedure

A solution of the polymer (∼10 mg) in 1 mL of THF (for **P1**) or dichloromethane (for **P4** and **P4-Pin**) was added to 3 equiv of *N*-hexylthymine and stirred for 5 min. The solvent was removed under reduced pressure, and the sample was dried under vacuum.

### Metal Coordination Procedure

A solution of the polymer (∼10 mg) in 3 mL of dichloromethane was added to 1 equiv of Pd-SCS-pincer, followed by 1 equiv of AgBF_4_ in 1 mL of acetonitrile. The mixture was stirred for 1 h with AgCl precipitating out of the solution. The mixture was filtered using a 0.45 μm syringe filter. The solvent was removed under reduced pressure, and the sample was dried under vacuum.

### Hydrogen-Bonding Disassembly

The **P1-Thy** sample (10 mg) in 1 mL of dichloromethane was precipitated in 50 mL of methanol, and the precipitated product was filtered and dried under vacuum.

### Metal Coordination Disassembly

One equivalent of triphenylphosphine was added to the polymer–pincer assembly (∼20 mg) in 4 mL of dichloromethane. The mixture was stirred for 1 h. The mixture was condensed under reduced pressure, and a large amount of acetonitrile was poured into the mixture, resulting in the precipitation of the polymer. The precipitation was repeated at least three times to fully remove the PPh_3_–Pin complexes. The polymer was obtained by filtration and dried under vacuum.

### Measurement of Association Constant *K*_a_

*K*_a_ was measured by ^1^H NMR titration experiments of **M1** or the target polymer (0.005 M based on repeat units) in CDCl_3_ or CD_2_Cl_2_ with a 0.10 M solution of *N*-hexylthymine. A shift of the DAP amide proton in the ^1^H NMR spectra was followed, and the *K*_a_ was calculated using the methods and program reported by Thordarson and co-workers.^42−44^

## Results and Discussion

### Synthesis of Monomers and Polymers

**Scheme 1:**
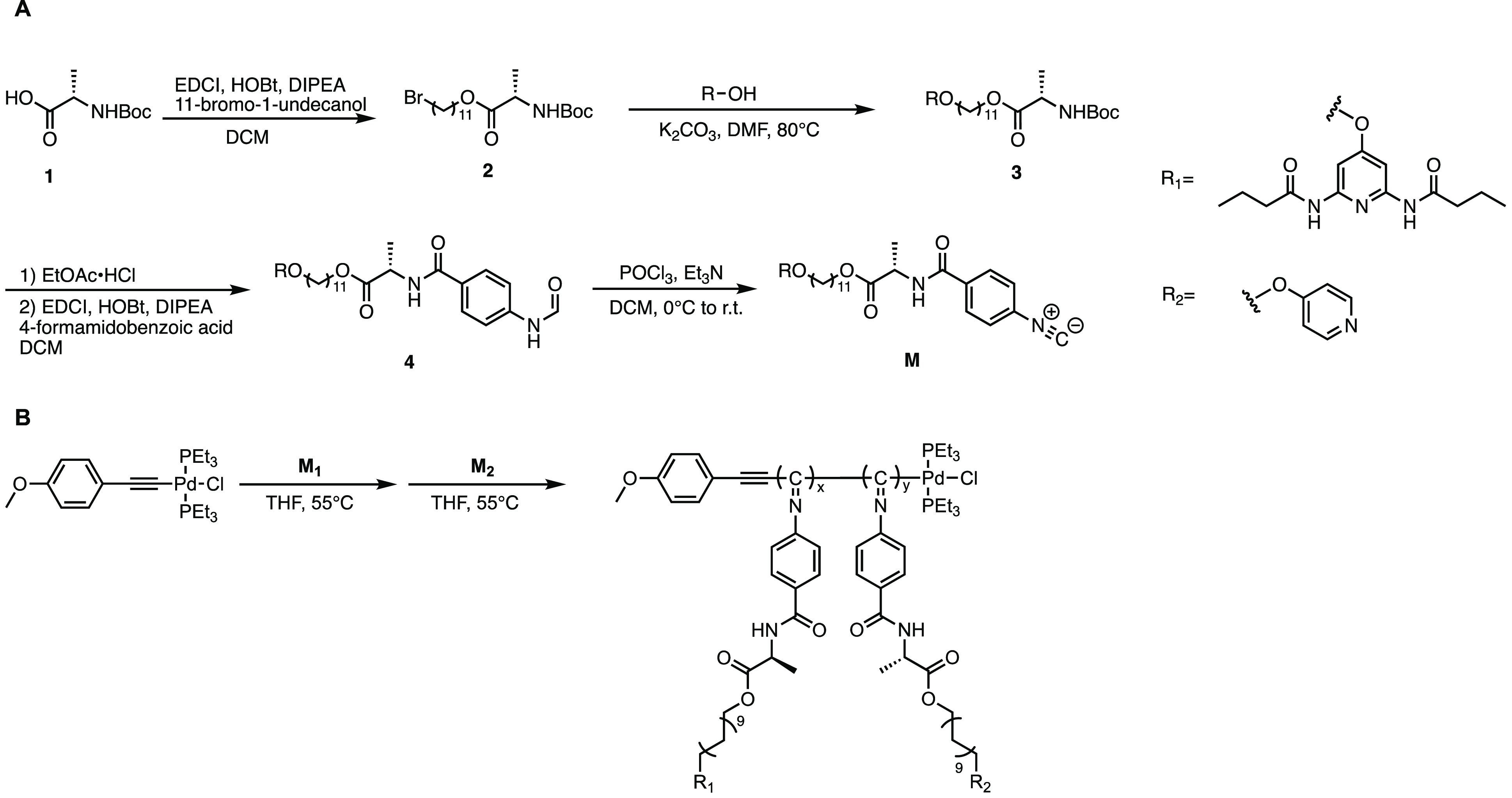
Synthetic Route of (A) Monomers DAP-NC (**M1**) and Py-NC (**M2**) and (B) Polymers

Our design uses two different supramolecular motifs: a hydrogen-bonding pair and a metal coordination pair. These two assemblies were chosen as they differ in their association strength under the same conditions, can be disassembled under orthogonal conditions, and allow for easy characterization via NMR spectroscopy. To eliminate any effects of monomer structure on the assembly behavior, we designed a modular synthetic route (Scheme 1) to obtain the monomers that only differ in their MRUs. The 4-hydroxyl-2,6-diaminopyridine motif was prepared according to the literature.^45^ Boc-protected l-alanine was incorporated into the monomer to install a chiral center, enabling the formation of helices of a preferred handedness upon polymerization. Protected amino acid **1** was first attached to an undecyl linker terminated with a substitutable bromine through esterification. Subsequently, the DAP and Py groups were attached to **2** by refluxing the reactants in DMF with K_2_CO_3_ for 2 days. The Boc-protecting group of **3** was removed with hydrochloric acid generated *in situ* using ethanol and acetyl chloride, and the generated ammonium compound was coupled to a previously synthesized 4-formamidobenzoic acid.^38^ The phenyl isocyanide monomers were obtained by dehydration of the formamide **4** using POCl_3_.

**Figure 2:**
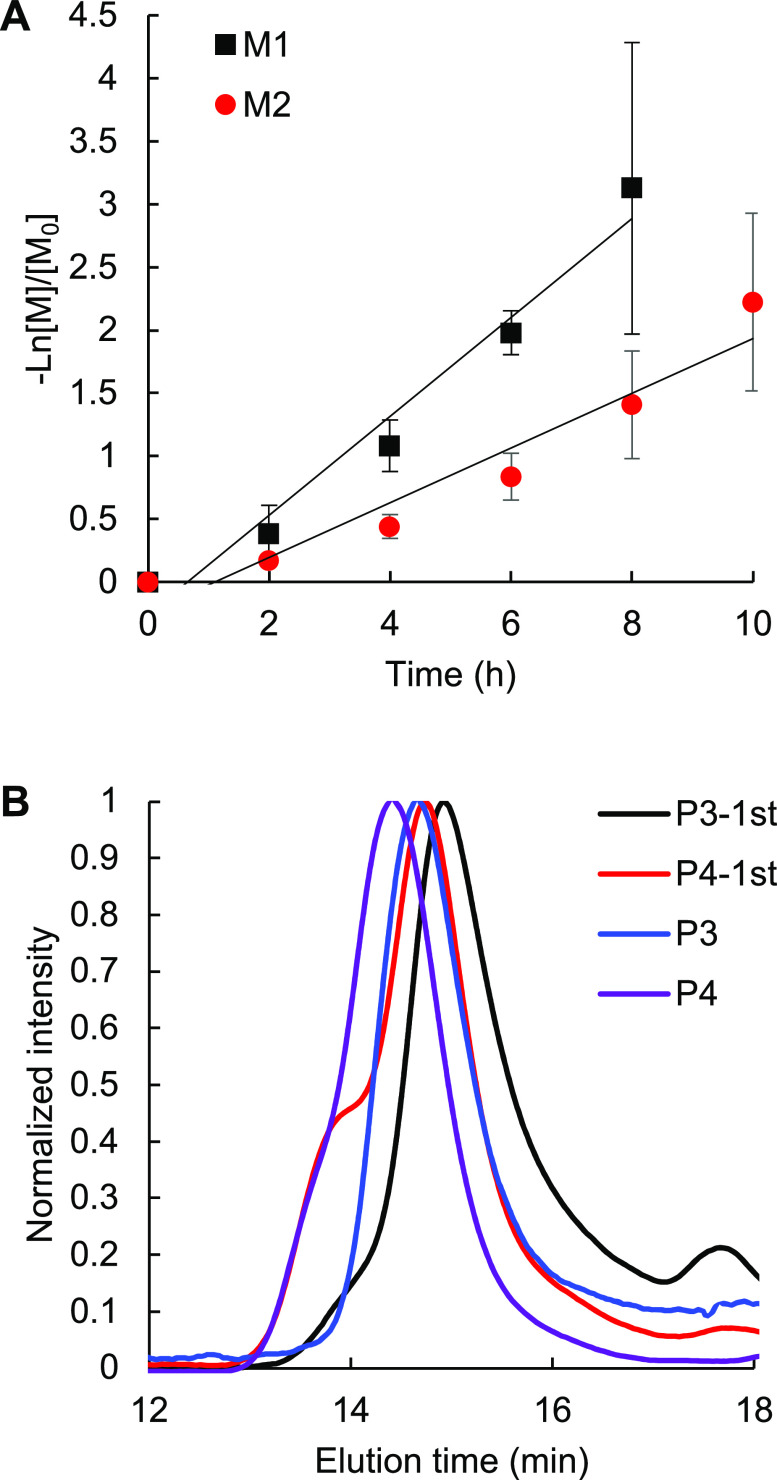
(A) Polymerization kinetic plots of **M1** (black) and **M2** (red). [M_0_] = 0.2 M, [M_0_]/[I] = 50 in THF at 55 °C. (B) GPC traces of sequential block copolymerization for **P3**-1st block (black), **P4**-1st block (red), **P3** (blue), and **P4** (purple).

The polymerization of the isocyanides was performed with a reported palladium–alkyne catalyst^19,46^ in THF at 55 °C. The polymer GPC characterization data are summarized in Table 1. Because of the different hydrodynamic radii in different solvents, the prepared polymers **P1** and **P2** exhibit different measured molecular weights in THF and DMF. DAP containing poly(isocyanide) **P1** (GPC traces, see Figures S37 and S38) exhibits poor solubility in dichloromethane and chloroform while THF, DMF, or a mixture of methanol and dichloromethane (15/85, v/v) dissolves the polymer. On the contrary, Py-containing poly(isocyanide) **P2** (GPC tracess see Figures S37 and S38) shows high solubility in dichloromethane, THF, and DMF. The polymerization kinetic plots of the monomers are shown in Figure 2A. The polymerization of **M1** shows first-order kinetics, which indicates a living polymerization. The polymerization rate also matches with reports from related monomers from the Wu group.^46^ This suggests that the polymerization is unaffected by the DAP side-chains. The polymerization of **M2** is slower than **M1**, which might be the result of competition for the palladium coordination sites between the pyridine groups and the triethylphosphine, and the isocyanide groups. With the different polymerization kinetic behaviors of the two monomers, we targeted the synthesis of block copolymers of **M1** and **M2**. **M1** and the palladium catalyst were stirred in THF at 55 °C for 24 h before **M2** was added to ensure the full consumption of **M1**. After the addition of **M2** to the reaction, the polymerization proceeded for an additional 48 h to ensure the full conversion of **M2**. We first targeted a block copolymer with a 30:30 **M1** to **M2** ratio. The obtained block copolymer is not fully soluble in dichloromethane and chloroform. To investigate the assembly behavior of the target block copolymer to complementary small molecules, high solubility of the polymer in a range of solvents is necessary. Therefore, we adjusted the ratio of **M1** to **M2** to 20:40 which generated a block copolymer fully soluble in halogenated solvents. GPC traces of **P3** and **P4** shifted to higher molecular weights compared to the first block which proved the successful block copolymerization (Figure 2B). The compositions and monomer ratios of the block copolymers were confirmed by ^1^H NMR spectroscopy (Figure S21). The integration of the DAP aromatic proton peak and the α-pyridyl proton peak has a ratio of 1:2 which is consistent with the targeted **M1** to **M2** ratio. The helical confirmation of all polymers was confirmed using circular dichroism (CD) spectroscopy. All polymers exhibit similar CD patterns (Figures S30, S31, and 8C,D) with a major negative Cotton effect at 365 nm that originates from the n−π* transition of the imine backbone. The negative signal of the absorption indicates a preferred left (*M*)-handedness of the synthesized poly(isocyanide)s. With all the polymers in hand, we investigated the supramolecular assembly of these polymers with complementary motifs.

**Table 1:** GPC Data of Polymers **P1–P4**

entry	[M_1_]/[M_2_]/[I]	*M*_n_ (kDa)	*Đ*	*M*_cal_ (Da)
**P1**^a^	30/0/1	7.9	1.22	19583
**P1**^b^	30/0/1	17.6	1.81	19583
**P2**^a^	0/30/1	4.1	1.29	14477
**P2**^b^	0/30/1	6.6	1.34	14477
**P3**^b^	30/30/1	20.3	1.45	33551
**P4**^b^	20/40/1	13.8	1.19	31849

a Eluent THF.

b Eluent DMF (with 0.03 M LiCl). Poly(styrene) standards.

### Hydrogen Bonding

**Figure 3:**
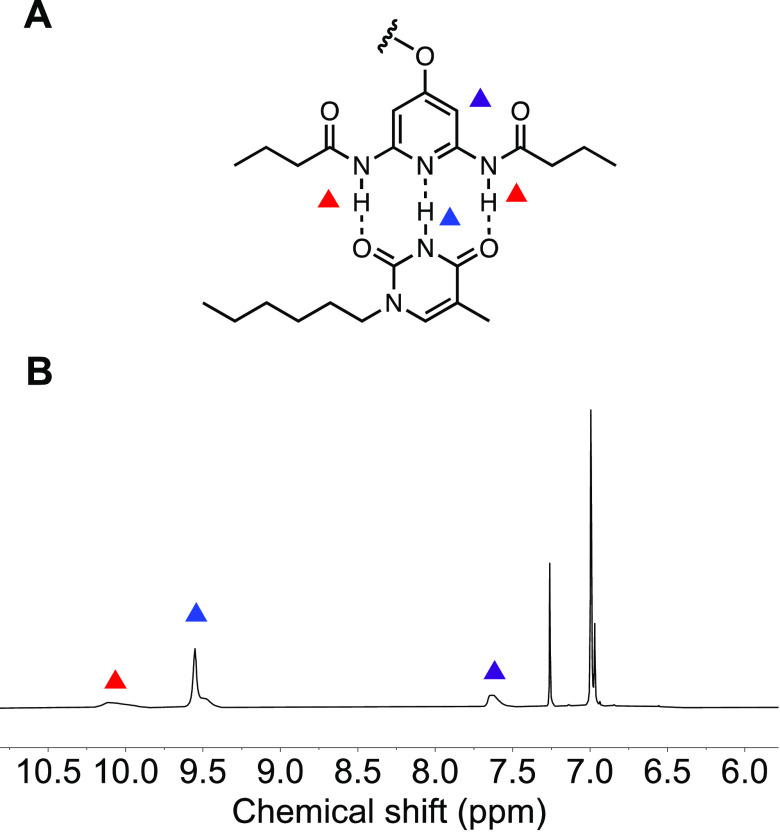
(A) Proton peaks assignment of the DAP-Thy pair. (B) ^1^H NMR spectrum of **P1-Thy** assembly in CDCl_3_.

Diaminopyridine and thymine are a DAD/ADA complementary hydrogen-bonding motif that has been widely explored in supramolecular chemistry and polymer science.^47−54^ We chose the DAP/Thy pair over other hydrogen-bonding motifs because of the ease of synthesis and relatively strong association constant (*K*_a_ = 10^2^–10^3^ M^–1^ in CDCl_3_).^47,55^ We first investigated the hydrogen bonding of the DAP-bearing monomer **M1** with *N*-hexylthymine prepared by refluxing thymine with *n*-bromohexane in acetonitrile. ^1^H NMR titration experiments in CDCl_3_ (Figure S25) proved the hydrogen bonding of **M1** to *N*-hexylthymine. The amide proton of DAP shifted downfield from a very broad signal at 7.50 to 10.01 ppm. Approximately 3 equiv of *N*-hexylthymine is required to realize the assembly equilibration to the DAP isocyanide monomer. The association constant *K*_a_ of 881 ± 267 M^–1^, measured from the ^1^H NMR titration experiments in CDCl_3_, matches with previously reported values of similar compounds.^39,40,47^ Next, we investigated the hydrogen bonding along the polymers. Because **P1** has poor solubility in chloroform, no association constant could be obtained in chloroform. To understand the effect of polymerization on the hydrogen-bonding ability of DAP with Thy, we performed ^1^H NMR titration experiments (Figures S26 and S27) of both **P1** and **M1** with *N*-hexylthymine in THF-*d*_8_. THF, a hydrogen-bond-accepting solvent, competes with the hydrogen bond acceptors of Thy and DAP. The DAP amide proton of **M1** shifted downfield from 7.50 ppm in CDCl_3_ to 8.75 ppm in THF-*d*_8_. This downfield shift indicates the formation of hydrogen bonds between DAP and THF-*d*_8_. As expected, the measured association constants of **M1** and *N*-hexylthymine in THF-*d*_8_ decreased an order of magnitude to 38 ± 7 M^–1^ from 881 ± 267 M^–1^ in CDCl_3_. The DAP amide proton peak of **P1** corresponds to the peak at 8.86 ppm, which is in the slightly lower field than **M1** in THF-*d*_8_. The *K*_a_ of **P1** and *N*-hexylthymine in THF-*d*_8_ decreased to 20 ± 5 M^–1^ compared with **M1** and *N*-hexylthymine. This weakened binding affinity of the polymer to Thy could be the result of limited accessibility of *N*-hexylthymine to the DAP tethered to the polymer backbone. Nonetheless, the *K*_a_ values of **P1** and **M1** to *N*-hexylthymine still have the same order of magnitude. Based on the monomer titration study (titration curve, see Figure S25), 3 equiv of *N*-hexylthymine (based on repeat units) was added to a **P1** solution in THF to study the effect of hydrogen bonding on the properties of **P1**. Interestingly, after THF was evaporated, the **P1-Thy** sample became soluble in dichloromethane and chloroform. It is noteworthy that the DAP amide proton of **P1-Thy** revealed itself at 10.10 ppm in CDCl_3_, which exhibited the same significant downfield shift as **M1** when complexed with *N*-hexylthymine (Figure 3).

### Metal Coordination

**Figure 4:**
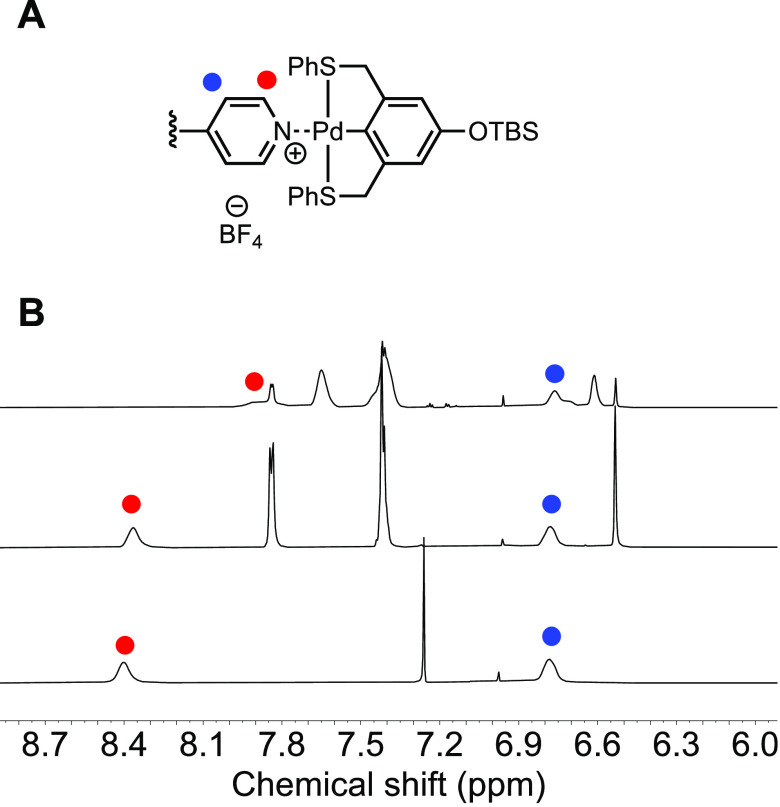
(A) Proton peaks assignment of the Py-Pin pair. (B) ^1^H NMR spectrum of **P2** (bottom) in CDCl_3_, **P2+Pin** physical mixture (middle), and **P2-Pin** assembly (top) in CD_2_Cl_2_.

**Figure 5:**
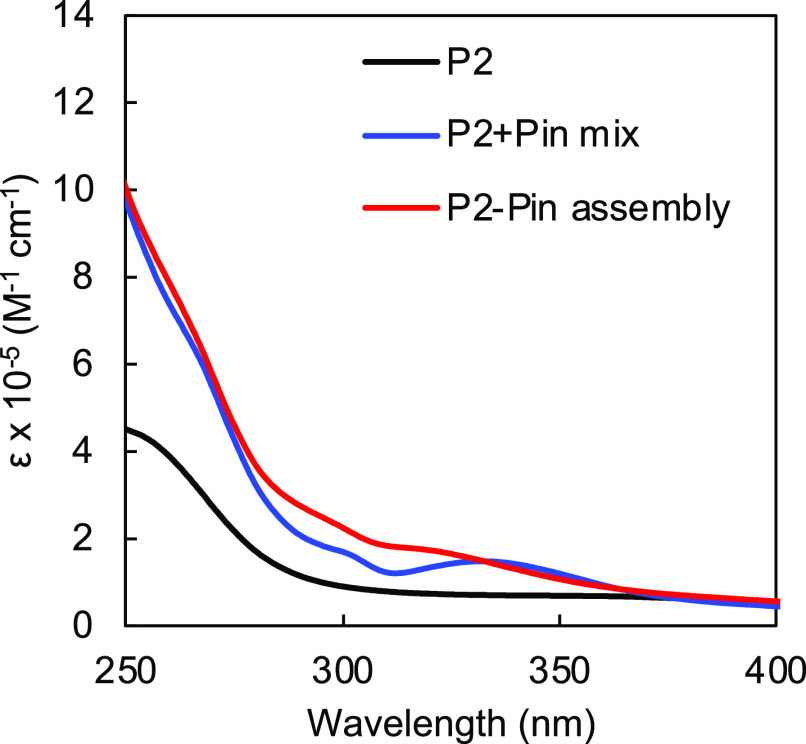
UV–vis spectrum of **P2** (black), **P2+Pin** physical mixture (blue), and **P2-Pin** assembly (red) in chloroform.

Pd-SCS-pincer is a well-known motif that binds to σ-donor ligands such as nitriles, pyridines, and phosphines. The Weck group has demonstrated numerous examples of polymer assemblies enabled by the Py–Pin metal coordination pair.^27,28,38−40^ To understand the assembly behavior of Pin to the pyridine-functionalized helical poly(isocyanide)s, we started with assembly experiments using the Py-containing homopolymer **P2**. The assembly of **P2** in dichloromethane was performed by the addition of 1 equiv of the Pd–SCS–pincer ligand to **P2** followed by the addition of 1 equiv of AgBF_4_ in acetonitrile. A white precipitate of AgCl formed upon the addition of AgBF_4_, indicating that the chlorine anion was removed from the Pin molecule. The assembled **P2-Pin**, however, formed aggregates in chloroform so dichloromethane-*d*_2_ was used as the solvent for NMR study. ^1^H NMR (Figure 4) and UV (Figure 5) spectroscopies both confirmed the successful assembly of the Pin unit with the pyridines of **P2**. As Figure 4 shows, the α-pyridine proton peak shifted upfield from 8.40 to 7.90 ppm and was broadened compared with the **P2** and **P2+Pin** physical mixture. This is the characteristic shift of the pyridine proton peak upon assembly with the Pin complex. Using UV–vis spectroscopy, we observed that the absorption peak at 335 nm of the physical mixture of **P2+Pin** shifted toward shorter wavelength around 315 nm upon addition of AgBF_4_. The observed NMR and UV–vis spectroscopic changes induced by the metal coordination matched with our previously reported data.^38,39^

### Orthogonal Supramolecular Assembly

**Figure 6:**
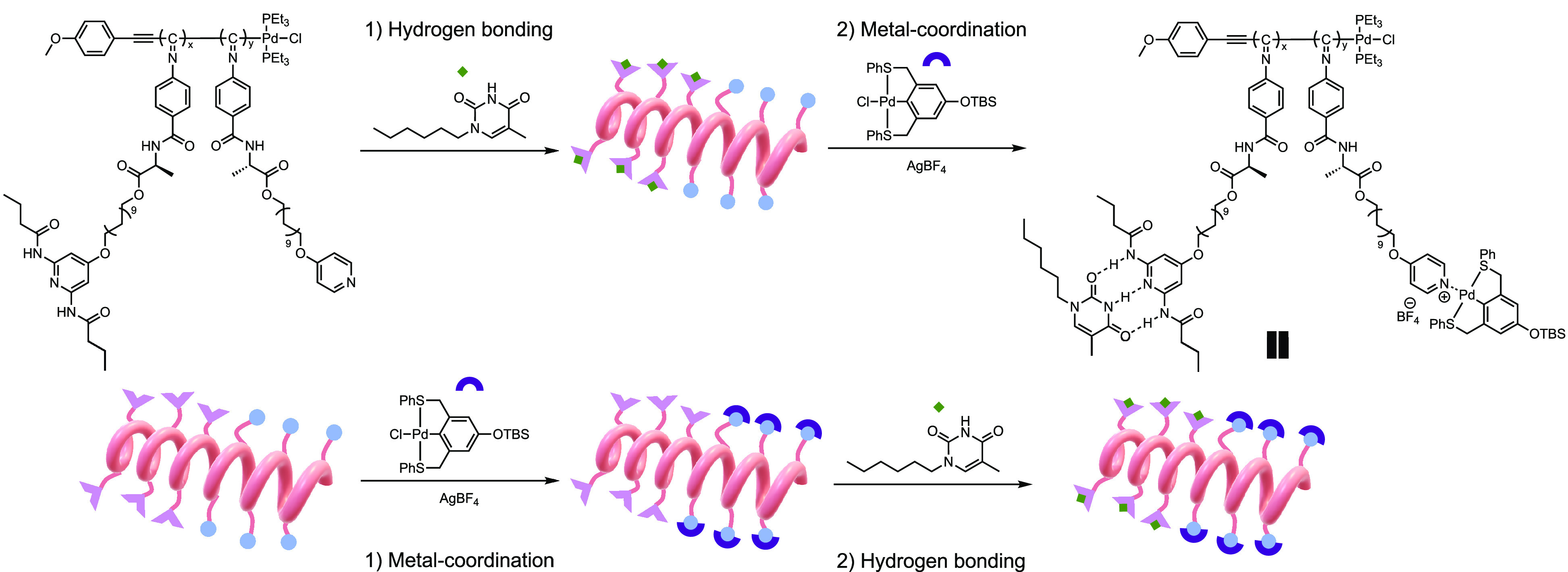
Schematic representation of stepwise supramolecular assembly in different orders.

After confirming the successful noncovalent assembly of Thy and Pin to their complementary MRUs along the side-chains of helical poly(isocyanide)s, we applied the two assembly motifs to the prepared diblock copolymer. To investigate whether they interfere with each other, we adapted two different strategies of assembly: hydrogen bonding followed by metal coordination or vice versa (Figure 6).

**Figure 7:**
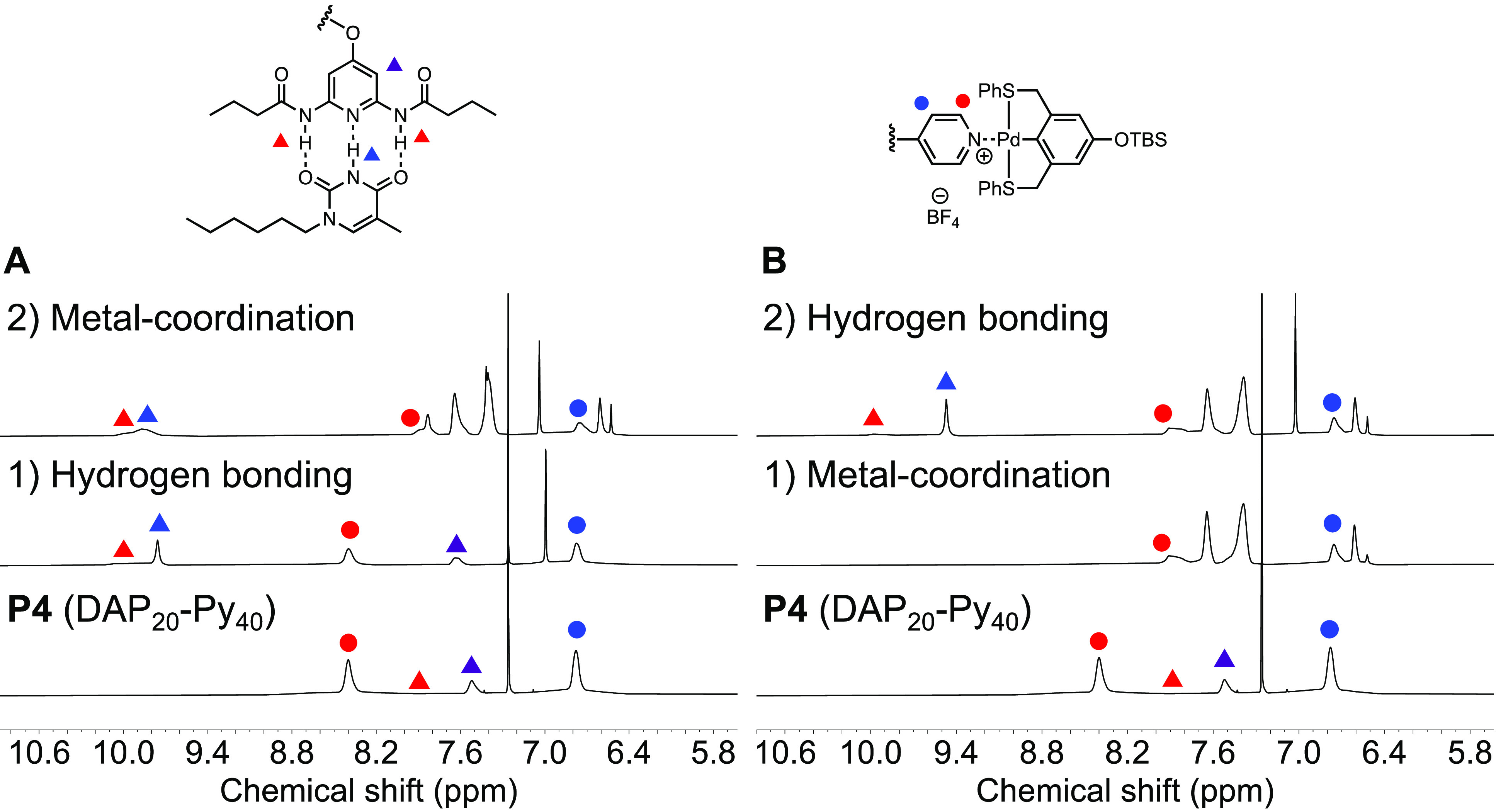
^1^H NMR spectra of (A) **P4** (bottom), **P4-Thy** (middle) in CDCl_3_, and **P4-Thy-Pin** (top) in CD_2_Cl_2_ and (B) **P4** (bottom) in CDCl_3_, **P4-Pin** (middle), and **P4-Pin-Thy** (top) in CD_2_Cl_2_.

**Figure 8:**
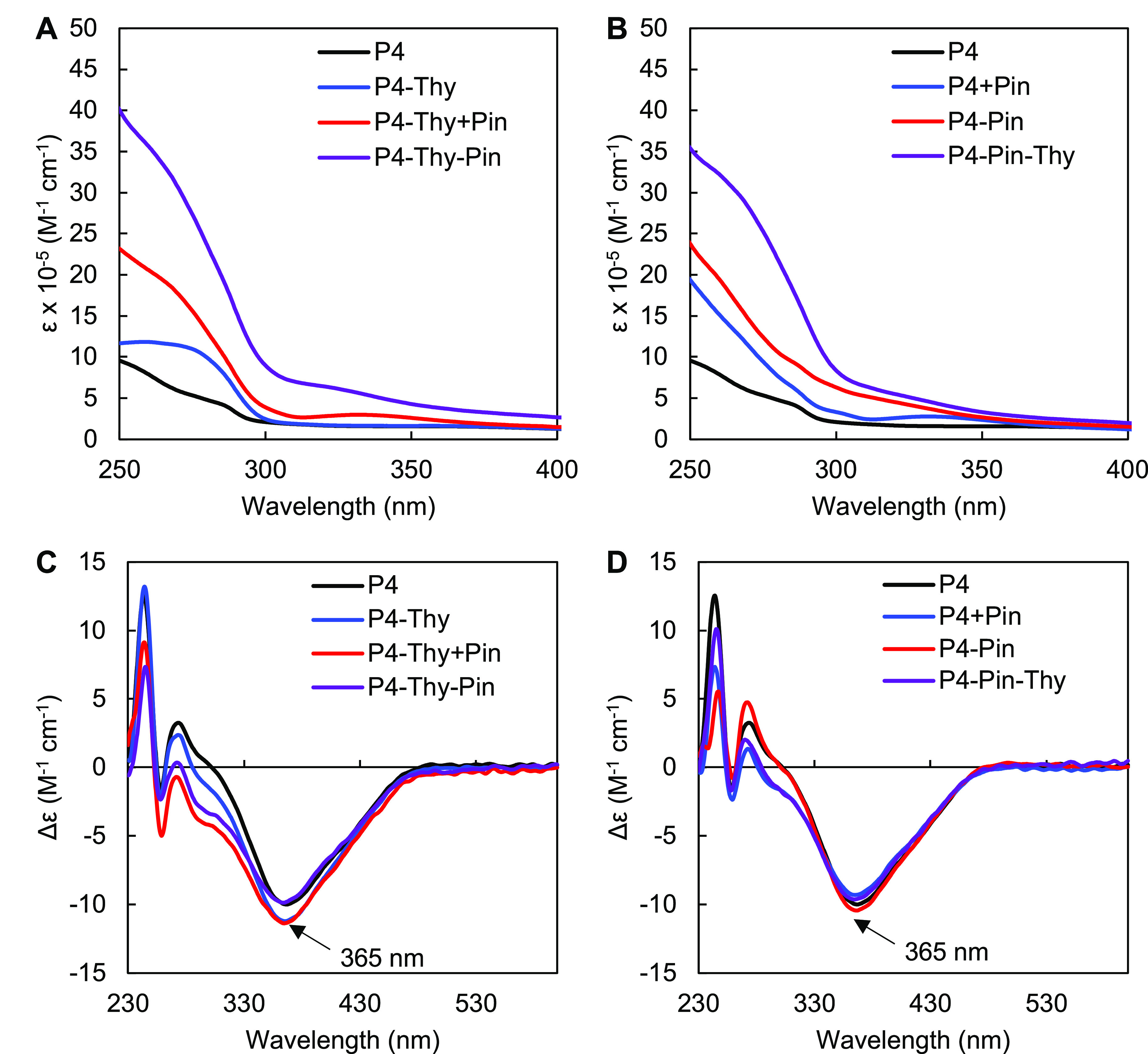
UV–vis (A, B) and CD (C, D) spectra of **P4**, **P4-Thy**, **P4-Thy+Pin** physical mixture, **P4-Thy-Pin** and **P4**, **P4+Pin** physical mixture, **P4-Pin**, and **P4-Pin-Thy** in chloroform.

**P4** was chosen over **P3** for the multistep assembly study because of its better solubility in chloroform and dichloromethane. First, **P4** was assembled with *N*-hexylthymine by adding 3 equiv of *N*-hexylthymine to **P4** solution in CDCl_3_. The DAP amide proton shifted downfield to 10 ppm while the Py proton signals remained intact, as shown by the ^1^H NMR spectrum in Figure 7A. The **P4-Thy** sample was then mixed with 1 equiv of Pd-SCS-pincer ligand in dichloromethane followed by the addition of 1 equiv of AgBF_4_ in acetonitrile. The Py–Pin assembly was confirmed by the upfield shift of the α-pyridine proton signal. The DAP amide proton of **P4-Thy-Pin** remained around 10 ppm but slightly broadened compared with the ^1^H NMR spectrum of **P4-Thy**. Similar to the **P2-Pin** assembly, a shift of the UV absorption from at 335 nm of the physical mixture **P4-Thy+Pin** to at 315 nm of the assembled sample was observed (Figure 8A).

Next, the assembly was performed in the reverse order with **P4** by attaching the Pd-SCS-pincer to the pyridines along **P4** first. An upfield shift of the α-pyridine proton peak to 7.90 ppm was observed in the ^1^H NMR spectrum in Figure 7B. A similar blue-shift of the UV absorption (Figure 8B) was observed which further confirmed the metal coordination step. **P4-Pin** was then combined with 3 equiv of *N*-hexylthymine to conclude the two-step assembly. The ^1^H NMR spectrum of **P4-Pin-Thy** in dichloromethane-*d*_2_ shows the characteristic downfield shift of the DAP amide proton to around 10 ppm, which is consistent with **P1-Thy** and **P4-Thy**. No changes of the pyridine signals in the ^1^H NMR spectrum were observed. To quantitatively understand whether the association ability of the DAP unit to Thy was impacted by the assembled Py-Pin moiety, we measured the *K*_a_ of **P4** and **P4-Pin** to *N*-hexylthymine using ^1^H NMR titration experiments (Figures S28 and S29). Both the original polymer **P4** and the metal-coordinated **P4-Pin** have similar *K*_a_ values (689 ± 151 M^–1^ for **P4** and 701 ± 293 M^–1^ for **P4-Pin**). These values decreased slightly but are still comparable with the *K*_a_ of **M1** and *N*-hexylthymine. This indicates that the metal coordination did not significantly affect the hydrogen bonding between DAP and Thy. From our previous study of DAP-Thy assembly on a poly(norbornene) backbone, we found that the *K*_a_ of the polymer decreased around 50% compared with the *K*_a_ of monomer.^40^ The different effects from polymerization of norbornene and isocyanide most likely originate from the different rigidities of the poly(norbornene) and the poly(isocyanide) backbones. Poly(isocyanide) has a rigid helical backbone that might present the DAP units more easily. Overall, this data strongly supports our hypothesis that DAP-Thy and Py-Pin do not interfere with each other during the assembly process and can be considered orthogonal MRUs.

### Effect of Supramolecular Assembly on the Helical Conformation

Helicity plays an important role in protein structure and function. To emulate the structure of protein using synthetic polymers, the handedness of helical polymer must be retained during the functionalization and assembly process. The helical conformations of **P1** and **P1-Thy** were confirmed using CD spectroscopy. Both CD spectra (Figure S30) exhibit a negative Cotton effect at 365 nm which is characteristic of left-handed helical poly(isocyanide)s. The pattern and intensity of the spectrum were not affected significantly by the hydrogen-bonding step. The CD spectrum of **P2** (Figure S31) shows a similar pattern to **P1** and contains a negative Cotton effect at 365 nm. The physical mixture of **P2** and **Pin** shall not change the helical conformation of **P2** as there is no strong interaction between the two components. The CD intensity differences shown in Figure S31 between **P2** and **P2+Pin** might be attributed to the error from the concentration calculations as the concentration was calculated based on the theoretical molecular weight of the polymer and the expected degree of polymerization in combination with the assumption of a 1:1 ratio of Py/Pin and full coordination of all pyridine sites. The assembly of **P2-Pin** exhibits almost the same CD spectrum as the physical mixture. This indicates that the helical conformation was not significantly affected by the metal coordination assembly.

To investigate how the two orthogonal interactions of hydrogen bonding and metal coordination would affect the secondary structure of poly(isocyanide)s along multistep assembly, the CD spectra of the block copolymer and assembled polymers of each step were recorded in chloroform and shown in Figure 8C,D. **P4** exhibits a similar CD pattern as **P1** and **P2** with a major negative Cotton effect at 365 nm. As the CD spectra show, **P4-Thy**, **P4-Thy+Pin**, **P4-Thy-Pin**, **P4+Pin**, **P4-Pin**, and **P4-Pin-Thy** all have similar CD features and intensities with only slight differences that most likely originate from concentration calculations and baseline differences. For all hydrogen-bonded assemblies at a CD concentration of micromolar level, the binding site saturation percentages are low (ca. 16% for **P4-Thy** and ca. 10% for **P4-Pin-Thy**) as calculated using the association constant *K*_a_ and the polymer concentration (5 μM for **P4** and 2.5 μM for **P4-Pin**). To further examine helical conformation at high binding site saturation over 90%, we performed CD measurements with a large excess of *N*-hexylthymine (0.014 M). *N*-Hexylthymine has no UV–vis absorption from 320 to 600 nm (Figure S35). Thus, it did not affect the recording of the CD spectra in this region where the major Cotton effect at 365 nm was still observed for all samples (Figure S36). Overall, the similarity of the CD spectra confirmed that the helical conformations of the poly(isocyanide)s were maintained during the multistep assembly regardless of assembly orders.

### Reversibility of the Assembly

Considering the noncovalent nature of hydrogen bonding and metal coordination, the reversibility of both DAP-Thy and Py-Pin assemblies was investigated. Hydrogen-bonding pairs can be disrupted using polar solvents that can compete with the hydrogen-bonding interactions. Here, the assembled *N*-hexylthymine molecules were removed by pouring methanol into the polymer solution to precipitate out the original polymer. The obtained polymer became insoluble in dichloromethane and requires a mixture of methanol and dichloromethane (15/85, v/v) to dissolve it. The complete removal of *N*-hexylthymine was further characterized by a ^1^H NMR spectrum in THF-*d*_8_ (Figure S22) which shows the complete absence of the proton peaks assigned to *N*-hexylthymine, and all signals in the spectrum shifted back to the original position of **P1**. The CD spectrum (Figure S32) of the disassembled polymer is the same as the preassembled polymer.

To disrupt the assembly of Pin with Py, we used a competing ligand, triphenylphosphine, that is known to coordinate more strongly to palladium than pyridine.^56^ The success of the ligand displacement experiment was verified using the **P2-Pin** system. The disruption of the Py-Pin pair was confirmed by the return of the α-pyridine proton signal to 8.40 ppm in the ^1^H NMR spectrum (Figure S23). The helical conformation was sustained with almost no changes as shown in the CD spectrum (Figure S33). Removal of the PPh_3_-Pin was realized by precipitation of the sample in acetonitrile and subsequent filtration. The regenerated **P2** has the same ^1^H NMR and CD spectra as the original **P2**. This disassembly process opens the pyridine sites for reassembly and reuse for other purposes.

**Figure 9:**
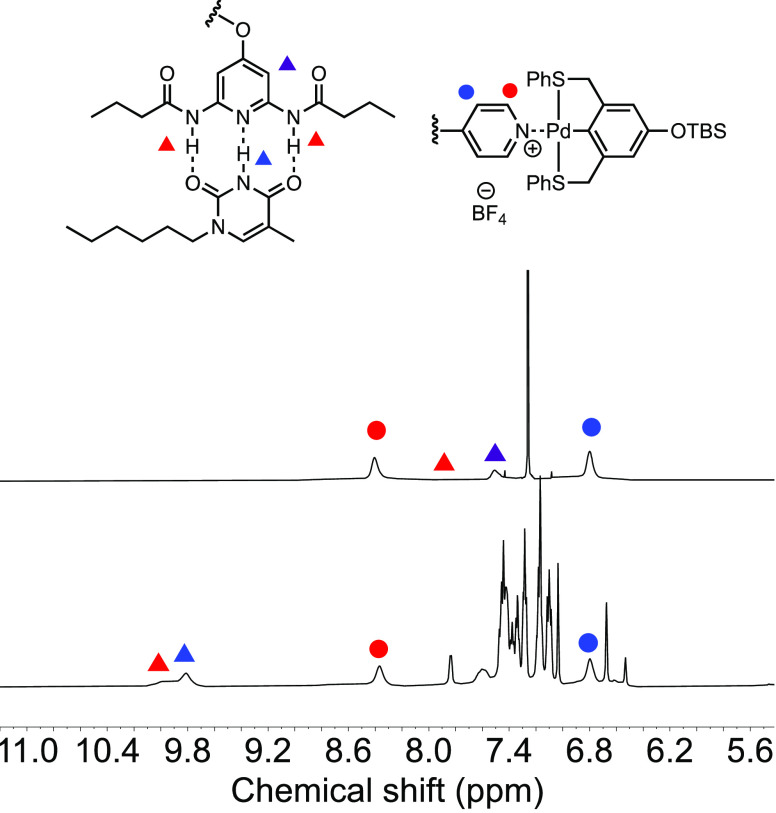
^1^H NMR spectra of **P4-Thy-Pin**+PPh_3_ (bottom) in CD_2_Cl_2_ and recycled **P4** (top) in CDCl_3_.

The same strategy was adapted for the disassembly of **P4-Thy-Pin** and **P4-Pin-Thy**. Because the hydrogen-bonding pair is easily broken, we targeted the selective disassembly of the metal coordination. The ^1^H NMR spectrum in Figure 9 shows (for full spectrum see Figure S24) the hydrogen-bonded DAP-Thy assembly remained intact after PPh_3_ was added to break the Py-Pin pair. The DAP amide proton peak stayed at around 10 ppm while the corresponding pyridine signal shifted back to its original position at 8.40 ppm. In order to remove PPh_3_-Pin, we adapted the same strategy to recycle **P2** by precipitation in acetonitrile. During the precipitation, the DAP-Thy assembly was also observed to disassemble as acetonitrile is a known hydrogen bond acceptor solvent. The ^1^H NMR spectrum of the precipitated polymer **P4** in Figure 9 shows that the *N*-hexylthymine proton peaks disappeared, and the DAP amide proton peak returned to 7.9 ppm from 10 ppm. This suggests that the polar acetonitrile removed the PPh_3_-Pin complexes as well as *N*-hexylthymine. The helical secondary structures of the block copolymers were retained after the hydrogen-bonding and metal coordination assemblies were reversed (for CD spectrum see Figure S34). Overall, the disassembly study proved that the two orthogonal assemblies are fully reversible; thus, the polymer can be regenerated and reused. This provides opportunities to tune the properties of the assembly accordingly and the reuse of the polymer scaffold which increases the dynamics of the helical building blocks.

## Conclusion

In conclusion, we have demonstrated an orthogonal supramolecular assembly strategy using hydrogen bonding and metal coordination that allows for easy functionalization of helical poly(isocyanide)s. The orthogonality of the assembly steps was demonstrated by stepwise functionalization in different orders with the final materials being the same by ^1^H NMR, UV, and CD spectroscopies. The disruption of the two assemblies and the regeneration of the original polymers evidenced the full reversibility of the assemblies. During the whole process of assembly and disassembly, the helical conformation remained intact as confirmed by CD spectroscopy. The presented strategy opens the possibility of using synthetic helical polymers as the core building blocks to obtain complicated folded polymeric architectures. In combination with other synthetic polymers with defined secondary structures, fully synthetic polymers with protein-like hierarchical structures are within reach. Moreover, the ability for orthogonal and full reversible assemblies anchored to helical polymers provides opportunities to create tunable and responsive materials.

## Supporting Information Available

Detailed synthetic procedures and characterizations for all monomers and supramolecular motifs; ^1^H NMR spectra, ^1^H NMR titration curves, CD spectra, and GPC traces for polymers (PDF)
