# Supplementary material for: Orthogonal Supramolecular Assemblies Using Side-Chain Functionalized Helical Poly(isocyanide)s
Source: Macromolecules. 2023 May 12;56(10):3507–16. doi: 10.1021/acs.macromol.2c02224 (PMC10210603; doi:10.1021/acs.macromol.2c02224)
Supplement: Supplementary file 1 — ma2c02224_si_001.pdf [file ma2c02224_si_001.pdf]

Orthogonal Supramolecular Assemblies Using Side-Chain Functionalized Helical Poly(isocyanide)s

Chengyuan Wang, Ru Deng, and Marcus Weck*

* E-mail: marcus.weck@nyu.edu

Department of Chemistry and Molecular Design Institute, New York University, New York, NY 10003, USA.

Table of Contents

Synthesis of monomers and supramolecular motifs 2

N,N'-(4-(benzyloxy)pyridine-2,6-diyl)dibutyramide 2

N,N'-(4-hydroxypyridine-2,6-diyl)dibutyramide (DAP-OH) 2

Compound 2 2

Compound 3-1 3

Compound 3-2 3

Compound 4-1 3

Compound 4-2 4

M1 4

M2 4

N-hexylthymine 5

(3,5-bis((phenylthio)methyl)phenoxy)(tert-butyl)dimethylsilane (Pd-SCS-pincer ligand) 5

Appendix 6

NMR spectra 6

^{1}H NMR Titration experiments 19

CD spectra 22

GPC traces 25

Synthesis of monomers and supramolecular motifs

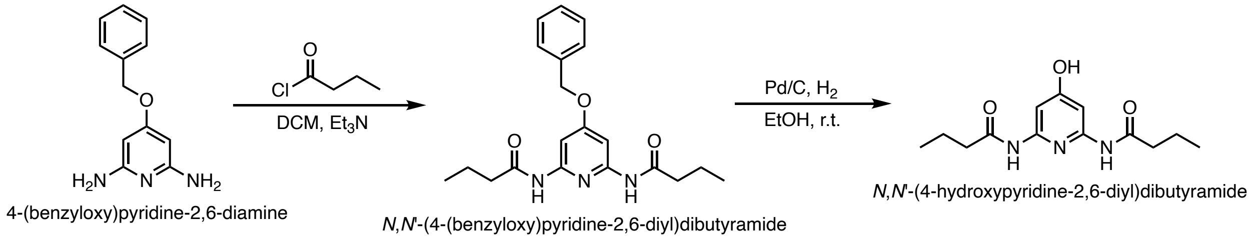

4-(Benzyloxy)pyridine-2,6-diamine was prepared according to a reported procedure.[1]

N,N′-(4-(benzyloxy)pyridine-2,6-diyl)dibutyramide

The compound was prepared via a modified method based on a literature procedure.[1] 4-(Benzyloxy)pyridine-2,6-diamine (2.50 g, 11.61 mmol) and triethylamine (3.53 g, 34.84 mmol) in dry DCM (100 mL) was cooled to 0 °C. Butyryl chloride (2.56 g, 24.04 mmol) was added slowly. The reaction was moved to ambient temperature after the addition and was stirred for three hours. The solvent was removed under reduced pressure and the crude was purified using silica gel column chromatography (hexane/EtOAc 2:1). The product was obtained as a colorless oil (1.91 g, yield 46%). ^{1}H NMR (500 MHz, $CDCl_3$, δ ppm): 7.65 (s, 2H), 7.60 (br s, 2H), 7.43 (m, 2H), 7.38 (m, 2H), 7.32 (m, 1H), 5.13 (s, 2H), 2.33 (t, J = 9 Hz, 4H), 1.74 (m, 4H), 1.00 (t, J = 9 Hz, 6H); ^{13}C NMR (126 MHz, $CDCl_3$, δ ppm): 171.6, 168.6, 150. 5, 135.8, 128.6, 128.2, 127.6, 96.4, 70.2, 39.7, 18.8, 13.7. HRMS(+ESI): $C_{20}H_{25}N_3O_3$, calculated 378.1788 $[M+Na]^+$, found 378.1806.

N,N′-(4-hydroxypyridine-2,6-diyl)dibutyramide (DAP-OH)

N,N′-(4-(benzyloxy)pyridine-2,6-diyl)dibutyramide (1.91 g, 5.37 mmol) was dissolved in ethanol (100 mL). 3 % Pd/C (700 mg) was added carefully to the flask. The mixture was purged with H_2 and stirred for four hours under H_2 atmosphere using a balloon. Pd/C was removed by filtration through celite. By removing the solvent under reduced pressure, the product was obtained as a sticky colorless oil (1.39 g, yield 98%). The product was used without further purification. ^{1}H NMR (600 MHz, DMSO-d_6, δ ppm): 9.75 (s, 2H) 7.27 (s, 2H), 2.34 (t, J = 9 Hz, 4H), 1.58 (m, 4H), 0.89 (t, J = 9 Hz, 6H); ^{13}C NMR (151 MHz, DMSO-d_6, δ ppm): 172.4, 167.0, 151.7, 97.3, 38.5, 18.9, 14.0. HRMS(+ESI): $C_{13}H_{19}N_3O_3$, calculated 288.1319 $[M+Na]^+$, found 288.1331.

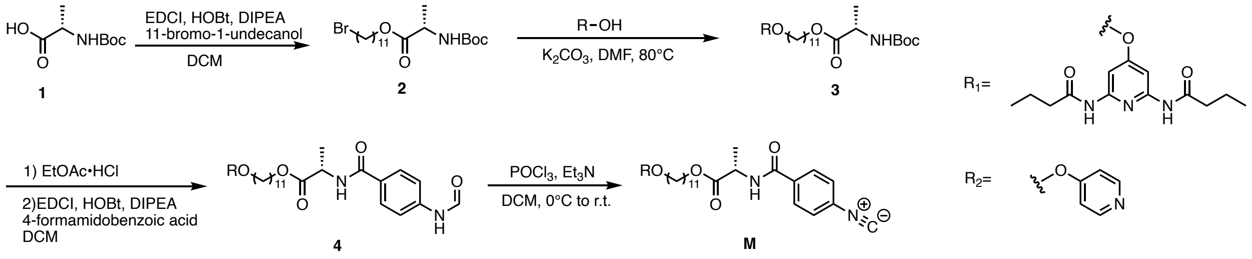

Compound 2

Boc-L-Ala-OH (8.29 g, 43.79 mmol) and 11-bromo-undecanol (10.00 g, 39.81 mmol) were dissolved in 150 mL DCM. DIPEA (7.72 g, 59.71 mmol), HOBt (9.14 g, 59.71 mmol), and EDCI

(11.45 g, 59.71 mmol) were added to the reaction mixture. The reaction was stirred overnight at room temperature. The reaction mixture was washed using 10% w/w citric acid, 10% w/w aqueous solution of $NaHCO_3$, and saturated brine. The organic layer was dried with Na_2SO_4 and the solvent was removed under reduced pressure. The compound was then purified using silica gel column chromatography (hexane/EtOAc 8:1, v/v) to obtain a colorless oil (7.32 g, yield 44%). ^{1}H NMR (600 MHz, $CDCl_3$, δ ppm): 5.04 (d, J = 6 Hz, 1H), 4.31 (mf, 1H), 4.09 (m, 2H), 3.41 (t, J = 9 Hz, 2H), 1.85 (m, 2H), 1.63 (m, 4H), 1.44 (s, 9H), 1.38 (d, J = 6 Hz, 3H), 1.35-1.20 (m, 14H); ^{13}C NMR (151 MHz, $CDCl_3$, δ ppm): 173.5, 155.1, 79.8, 65.5, 49.3, 34.1, 32.8, 29.43, 29.40, 29.2, 28.8, 28.5, 28.3, 28.2, 25.8, 18.8. HRMS(+ESI): $C_{19}H_{36}BrNO_4$, calculated 444.1720 $[M+Na]^+$, found 444.1725.

Compound 3-1

A suspension of 4-hydroxydiacetylaminopyridine (400 mg, 1.51 mmol), K_2CO_3 (420 mg, 3.02 mmol) in DMF (25 mL) was refluxed at 80 °C for 20 minutes. Compound **2** (670 mg, 1.58 mmol) was added to the suspension. The reaction was stirred for 24 hours at 80 °C. The solvent was removed under reduced pressure. The crude was dissolved in DI water and extracted using DCM (100 mL x 3). The organic phase was combined and dried with Na_2SO_4. The product was purified using silica gel column chromatography (hexane/EtOAc 2:1, v/v) to obtain a colorless oil (0.50 g, yield 55%). ^{1}H NMR (500 MHz, $CDCl_3$, δ ppm): 7.75 (br s, 2H), 7.53 (s, 2H), 5.13 (br s, 1H), 4.31 (m, 1H), 4.12 (t, J = 7.5 Hz, 2H), 4.02 (t, J = 5 Hz, 2H), 2.33 (t, J = 7.5 Hz, 4H), 1.76-1.71 (m, 4H + 2H), 1.63 (m, 2H), 1.44 (s, 9H), 1.37 (d, J = 10 Hz, 3H), 1.35-1.20 (m, 14H), 0.99 (t, J = 7.5 Hz, 6H); ^{13}C NMR (151 MHz, $CDCl_3$, δ ppm): 173.6, 171.9, 169.2, 155.2, 150.2, 96.0, 79. 8, 68.6, 65.6, 49.3, 39.6, 35.1, 29.4, 29.4, 29.2, 28.8, 28.5, 28.3, 25.81, 25.80, 18.8, 18.7, 13.7. HRMS(+ESI): $C_{32}H_{54}N_4O_7$, calculated 629.3885 $[M+Na]^+$, found 629.3889.

Compound 3-2

Compound **3-2** was prepared in a similar procedure to **3-1** as a colorless oil (yield 41%). ^{1}H NMR (600 MHz, $CDCl_3$, δ ppm): 8.43 (d, J = 6 Hz, 2H), 6.84 (d, J = 6 Hz, 2H), 5.06 (d, J = 6 Hz, 1H), 4.29 (m, 1H), 4.12 (m, 2H), 4.02 (t, J = 6 Hz, 2H), 1.80 (m, 2H), 1.63 (m, 2H), 1.44 (s, 9H), 1.38 (d, J = 6 Hz, 3H), 1.35-1.25 (m, 14H); ^{13}C NMR (151 MHz, $CDCl_3$, δ ppm): 173.5, 165.8, 155.1, 149.9, 110.6, 79. 8, 68.3, 65. 5, 49. 5, 29.5, 29.4, 29.3, 29.2, 28.8, 28.5, 28.3, 25.9, 25.8, 18.2. HRMS(+ESI): $C_{24}H_{40}N_2O_5$, calculated 459.2829 $[M+Na]^+$, found 459.2810.

Compound 4-1

Compound **3-1** (500 mg, 0.82 mmol) dissolved in ethyl acetate (5 mL) was added EtOAc·HCl mixture prepared from ethanol (31.4 mL) and acetyl chloride (22.9 mL). The reaction was stirred for 3 hours until the deprotection was fully complete. The solvent was removed under reduced pressure. The sample was dissolved in DCM and the solvent was removed to further get rid of excess HCl. The ammonia salt was dissolved in DCM. DIPEA (530 mg, 4.12 mmol), EDCI (320 mg, 1.65 mmol), HOBt (220 mg, 1.65 mmol) were added to the solution. The reaction was stirred at ambient temperature for 16 hours. The reaction mixture was washed using 10% w/w citric acid, 10% w/w aqueous solution of $NaHCO_3$, and saturated brine. The organic layer was dried with Na_2SO_4 and the solvent was removed under reduced pressure. The compound was then purified using silica gel column chromatography (hexane/EtOAc 2:1, v/v) to obtain a white solid (0.40 g, yield 74%). The aromatic region of the NMR spectrum of this compound became complicated by the formamide isomers[2] and thus the compound was used in the next step without further

purification and characterization after the mass spectroscopy confirmed the compound. ^{1}H NMR (600 MHz, $CDCl_3$, δ ppm): 8.81 (d, J = 11.4 Hz, 0.35 H), 8.42 (s, 0.65H), 8.20-7.40 (br m, 7.3H) 7.12 (d, J = 8.4 Hz, 0.7H), 6.77 (br s, 1H), 4.77 (m, 1H), 4.18 (m, 2H), 4.01 (m, 2H), 2.34 (m, 4H), 1.78-1.70 (m, 4H + 2H), 1.66 (m, 2H), 1.53 (d, J = 6 Hz, 3H), 1.43-1.25 (m, 14H), 0.99 (m, 6H); ^{13}C NMR (151 MHz, $CDCl_3$, δ ppm): 173.3, 171.7, 169.1, 159.1, 150.3, 129.8, 129.0, 128.2, 119.4, 117.7, 96.1, 68.6, 65.9, 65.8, 48.7, 39.7, 29.4, 29.36, 29.31, 29.24, 29.16, 29.11, 29.07, 29.0, 28.9, 28.8, 28.49, 28.47, 25.81, 25.78, 25.7, 18.7, 13.7. HRMS(+ESI): $C_{35}H_{51}N_5O_7$, calculated 676.3681 $[M+Na]^+$, found 676.3684.

Compound 4-2

Compound **4-2** was prepared in a similar procedure to **4-1** as a white solid (yield 87%). ^{1}H NMR (600 MHz, $CDCl_3$, δ ppm): 8.80 (d, J = 10.8 Hz, 0.35H), 8.41 (m, 2H + 0.65 H), 8.09 (d, J = 10.8 Hz, 0.35H), 8.06 (s, 0.65H), 7.82 (d, J = 8.4 Hz, 0.7H), 7.78 (d, J = 8.4 Hz, 1.3H), 7.64 (d, J = 8.4 Hz, 1.3H), 7.13 (d, J = 8.4 Hz, 0.7H), 6.81(m, 2H), 6.78 (d, J = 6.6 Hz, 1H), 4.77 (m, 1H), 4.17 (m, 2H), 4.00 (t, J = 9 Hz, 2H), 1.79 (m, 2H), 1.66 (m, 2H), 1.52 (d, J = 6 Hz, 3H), 1.44 (m, 2H), 1.35-1.25 (m, 12H); ^{13}C NMR (151 MHz, $CDCl_3$, δ ppm): 173.4, 166.0, 165.7, 165.4, 161.6, 159.0, 150.7, 150.6, 140.2, 139.9, 130.4, 129.8, 129.0, 128.2, 119.4, 117.7, 110.4, 68.0, 67.98, 65.9, 65.8, 48.7, 48.6, 29.48, 29.46, 29.43, 29.28, 29.26, 29.17, 29.15, 28.85, 28.84, 28.5, 25.9, 25.80, 25.79, 18.76, 18.74. HRMS(+ESI): $C_{27}H_{37}N_3O_5$, calculated 506.2625 $[M+Na]^+$, found 506.2628.

M1

Compound **4-1** (200 mg, 0.306 mmol) and triethylamine (155 mg, 1.529 mmol) were dissolved in 20 mL dry DCM and cooled to 0 °C. $POCl_3$ (70 mg, 0.459 mmol) was added using a syringe at once. The reaction was stirred for two hours at 0 °C and then moved to ambient temperature and stirred for 16 hours. The reaction was quenched using 10% w/w aqueous solution of $NaHCO_3$ and was washed using 10% w/w aqueous solution of $NaHCO_3$ and brine. The organic phase was dried with Na_2SO_4. The product was purified using silica gel column chromatography (hexane/EtOAc 3:2, v/v) to obtain a white sticky solid (162 mg, yield 84%). ^{1}H NMR (600 MHz, $CDCl_3$, δ ppm): 7.85 (d, J = 6 Hz, 2H), 7.53 (br s, 2H), 7.52 (s, 2H), 7.45 (d, J = 6 Hz, 2H), 6.83 (d, J = 12 Hz, 1H), 4.76 (m, 1H), 4.18 (m, 2H), 4.02 (t, J = 6 Hz, 2H), 2.33 (t, J = 6 Hz, 4H), 1.78-1.70 (m, 4H + 2H), 1.66 (m, 2H), 1.53 (d, J = 6 Hz, 3H), 1.42 (m, 2H), 1.38-1.20 (m, 12H), 0.99 (t, J = 6 Hz, 6H); ^{13}C NMR (151 MHz, $CDCl_3$, δ ppm): 173.1, 171.5, 169.1, 166.6, 165.0, 150.4, 134.8, 129.0, 128.4, 126.7, 96.1, 77.2, 77.0, 76.8, 68.5, 66.0, 48.8, 39.7, 29.42, 29.40, 29.36, 29.19, 29.13, 28.9, 28.5, 25.8, 25.77, 18.7, 18.6, 13.7. HRMS(+ESI): $C_{35}H_{49}N_5O_6$, calculated 659.3575 $[M+Na]^+$, found 659.3589.

M2

M2 was prepared using a similar procedure to **M1** as a white solid (yield 64%). ^{1}H NMR (600 MHz, $CDCl_3$, δ ppm): 8.41 (dd, J_1 = 4.8 Hz, J_2 = 1.2 Hz, 2H), 7.85 (d, J = 6 Hz, 2H), 7.45 (d, J = 6 Hz, 2H), 6.85 (d, J = 6 Hz, 1H), 6.80 (dd, J_1 = 4.8 Hz, J_2 = 1.8 Hz, 2H), 4.76 (m, 1H), 4.18(m, 2H), 4.00(t, J = 6 Hz, 2H), 1.79 (m, 2H), 1.66 (m, 2H), 1.53 (d, J = 6 Hz, 3H), 1.45 (m, 2H), 1.38-1.26 (m, 12H); ^{13}C NMR (151 MHz, $CDCl_3$, δ ppm): 173.13, 166. 7, 165.3, 165.0, 150.8, 134.8, 128.4, 126.7, 110.4, 67.9, 66.0, 48.8, 29.48, 29.46, 29.4, 29.3, 29.2, 28.9, 28.5, 25.9, 25.80, 18.7. HRMS(+ESI): $C_{27}H_{35}N_3O_4$, calculated 466.2700 $[M+H]^+$, found 466.2710.

N-hexylthymine

Thymine (5.00 g, 39.65 mmol), 6-bromohexane (3.27 g, 19.82 mmol) and K_2CO_3 (5.48 g, 39.65 mmol) in DMF (120 mL) was refluxed at 50 °C for 24 hours. The solvent was removed under reduced pressure. The crude was dissolved in DI water and extracted using DCM (100 mL x 3). The organic phase was combined and dried using Na_2SO_4. The product was obtained from silica gel column chromatography (EtOAc/hexane 2:1) and recrystallization in chloroform and hexane as a white solid (1.45 g, yield 17%). ^1H NMR (600 MHz, $CDCl_3$, δ ppm): 8.22 (s, 1H), 6.97 (d, J = 1.2 Hz, 1H), 3.68 (t, J = 6 Hz, 2H), 1.92 (d, J = 1.2 Hz, 3H), 1.67 (m, 2H), 1.35-1.28 (m, 6H), 0.89 (t, J = 6 Hz, 3H); ^{13}C NMR (151 MHz, $CDCl_3$, δ ppm): 163. 9, 150.6, 110.5, 48.6, 31.4, 29.08, 26.1, 22.5, 143.0, 12.4. HRMS(+ESI): $C_{11}H_{18}N_2O_2$, calculated 211.1441 $[M+H]^+$, found 211.1449.

(3,5-bis((phenylthio)methyl)phenoxy)(*tert*-butyl)dimethylsilane (Pd-SCS-pincer ligand)

The Pd-SCS-pincer compound was prepared according to a reported procedure.[3]

Appendix

NMR spectra

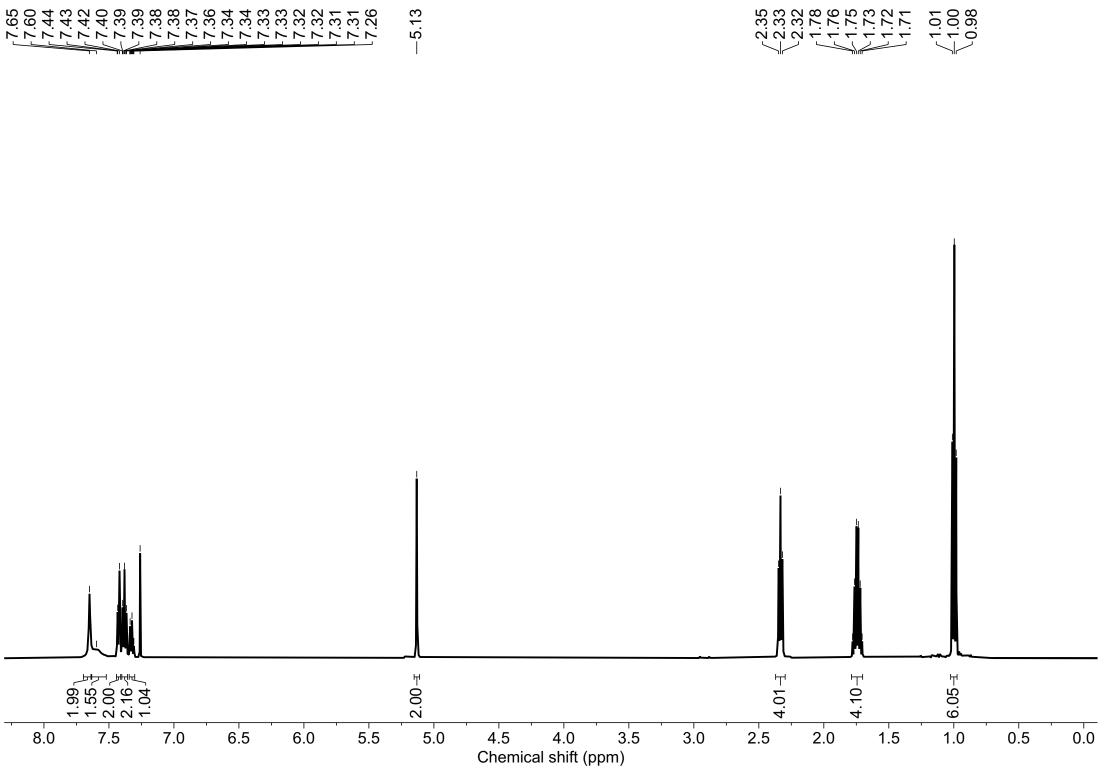

Figure S1. ^{1}H NMR spectrum of ***N,N'*-(4-(benzyloxy)pyridine-2,6-diyl)dibutyramide** in $CDCl_3$ (500 MHz).

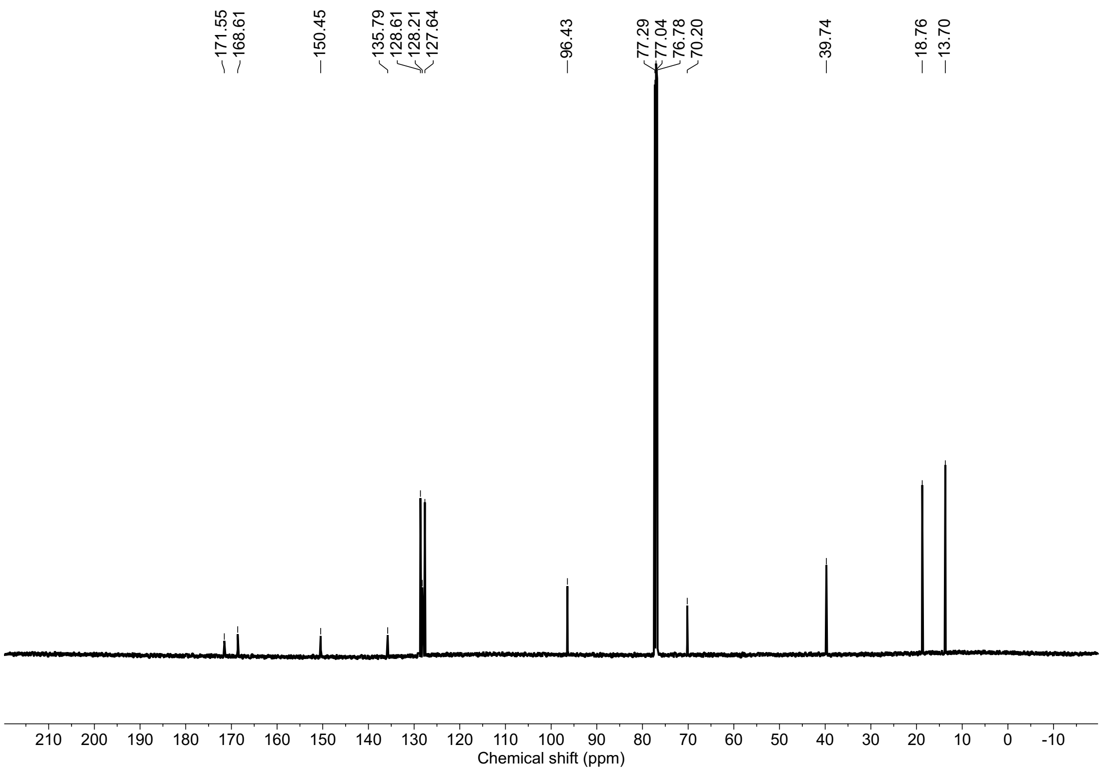

Figure S2. ^{13}C NMR spectrum of ***N,N'*-(4-(benzyloxy)pyridine-2,6-diyl)dibutyramide** in $CDCl_3$ (126 MHz).

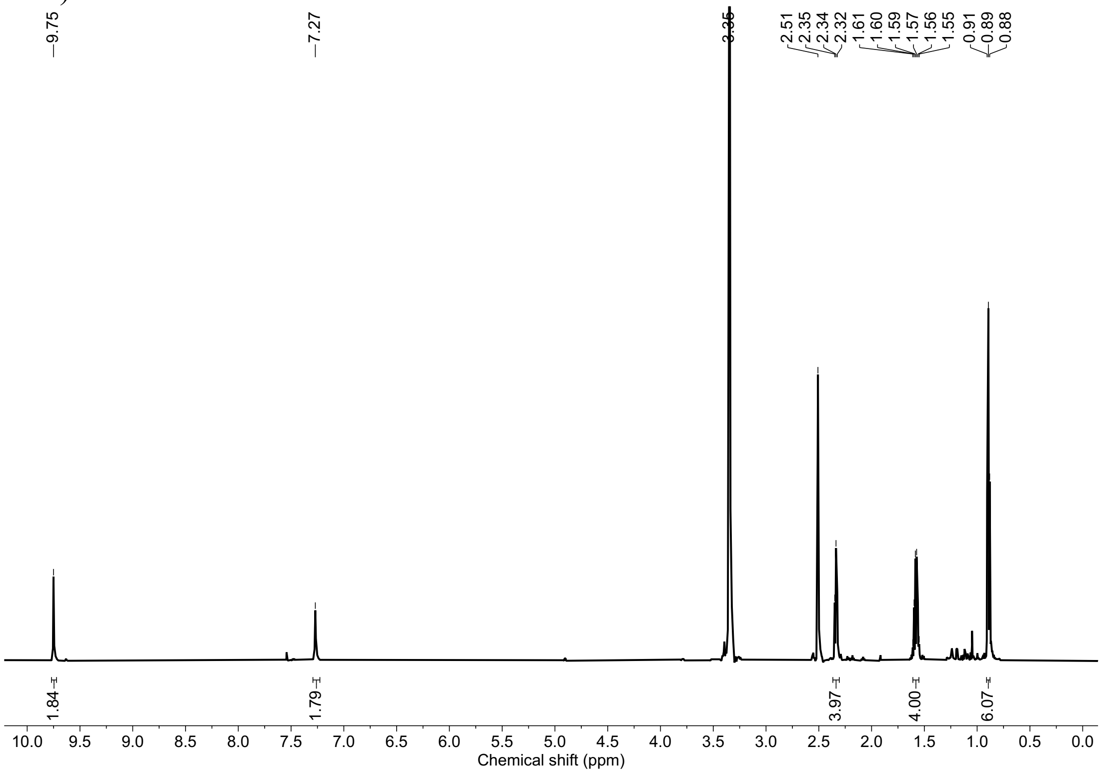

Figure S3. ^{1}H NMR of ***N,N'*-(4-hydroxypyridine-2,6-diyl)dibutyramide** in DMSO-d_6 (600 MHz).

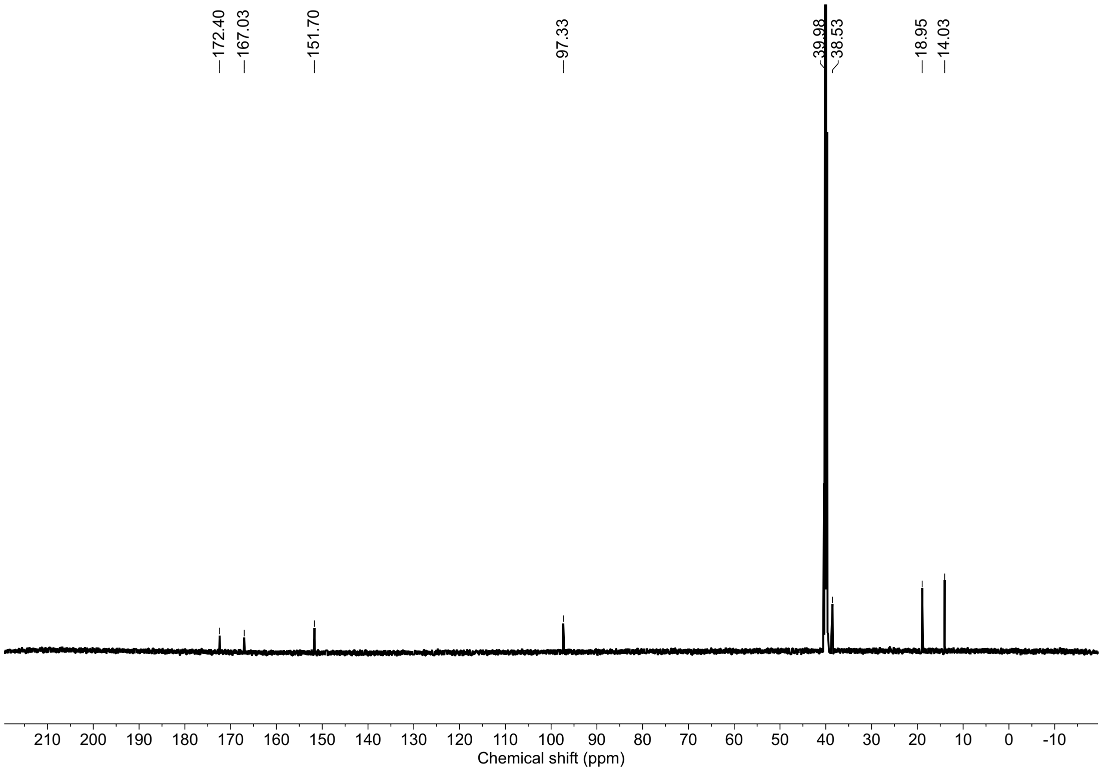

Figure S4. ^{13}C NMR spectrum of ***N,N′*-(4-hydroxypyridine-2,6-diyl)dibutyramide** in DMSO-d_6 (151 MHz).

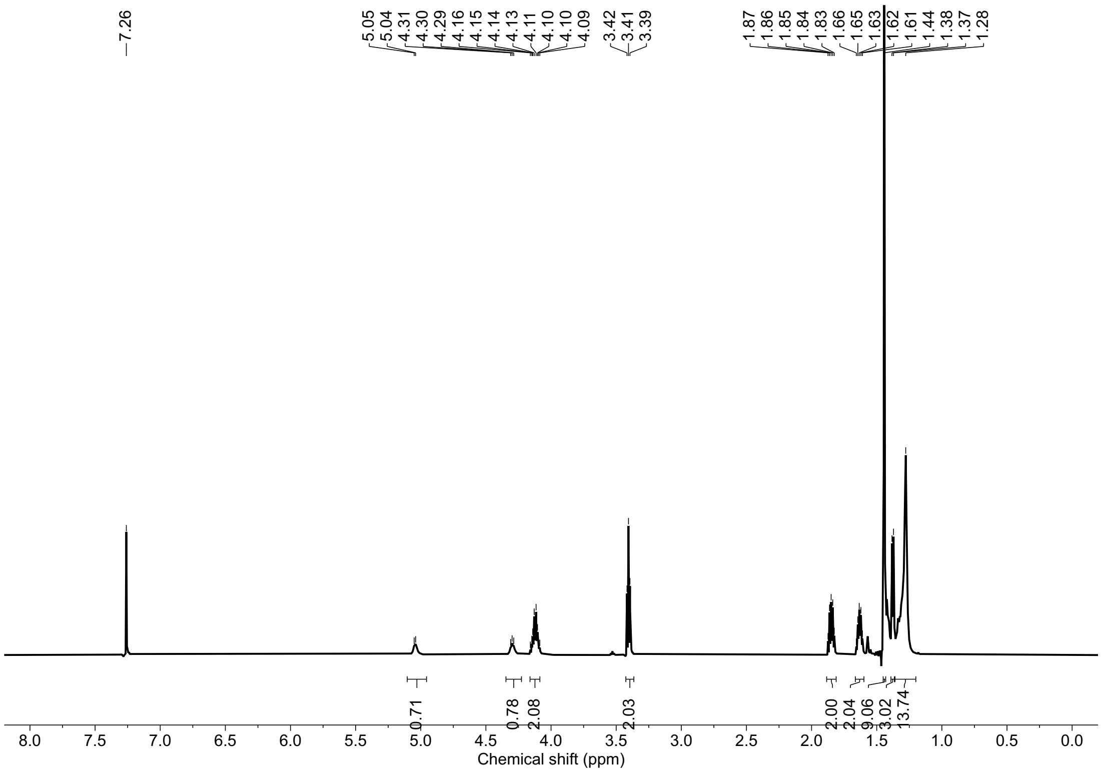

Figure S5. ^{1}H NMR spectrum of **2** in $CDCl_3$ (600 MHz).

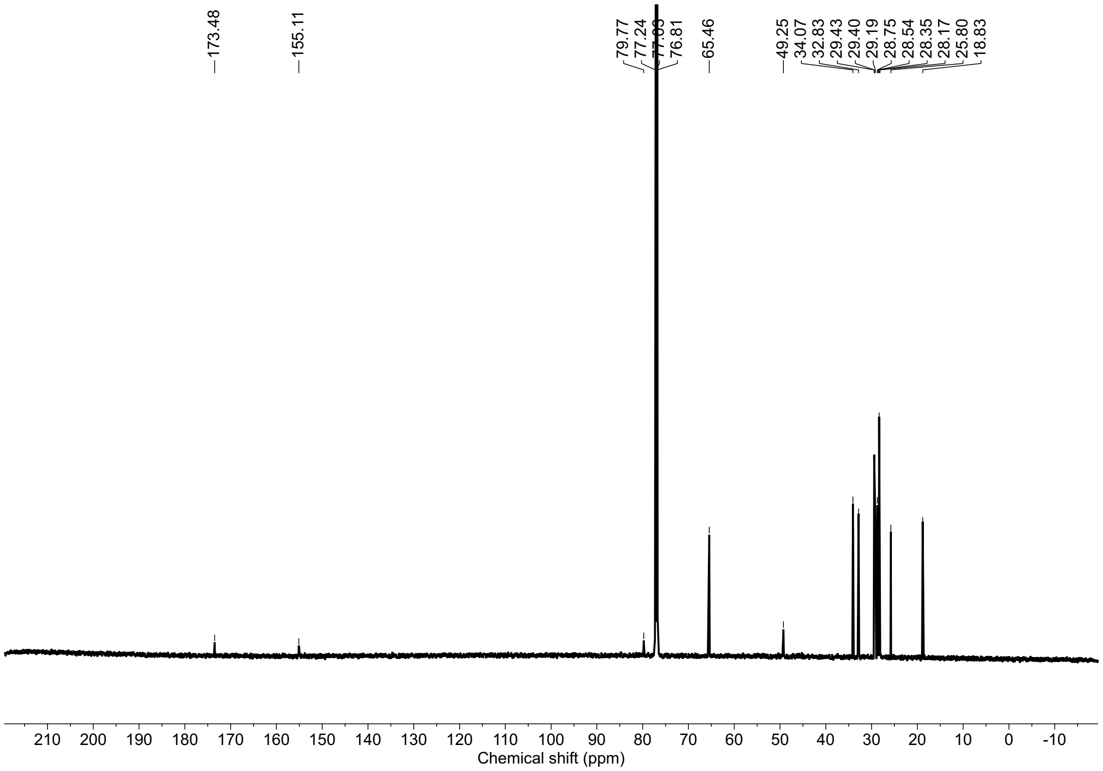

Figure S6. ^{13}C NMR spectrum of **2** in $CDCl_3$ (151 MHz).

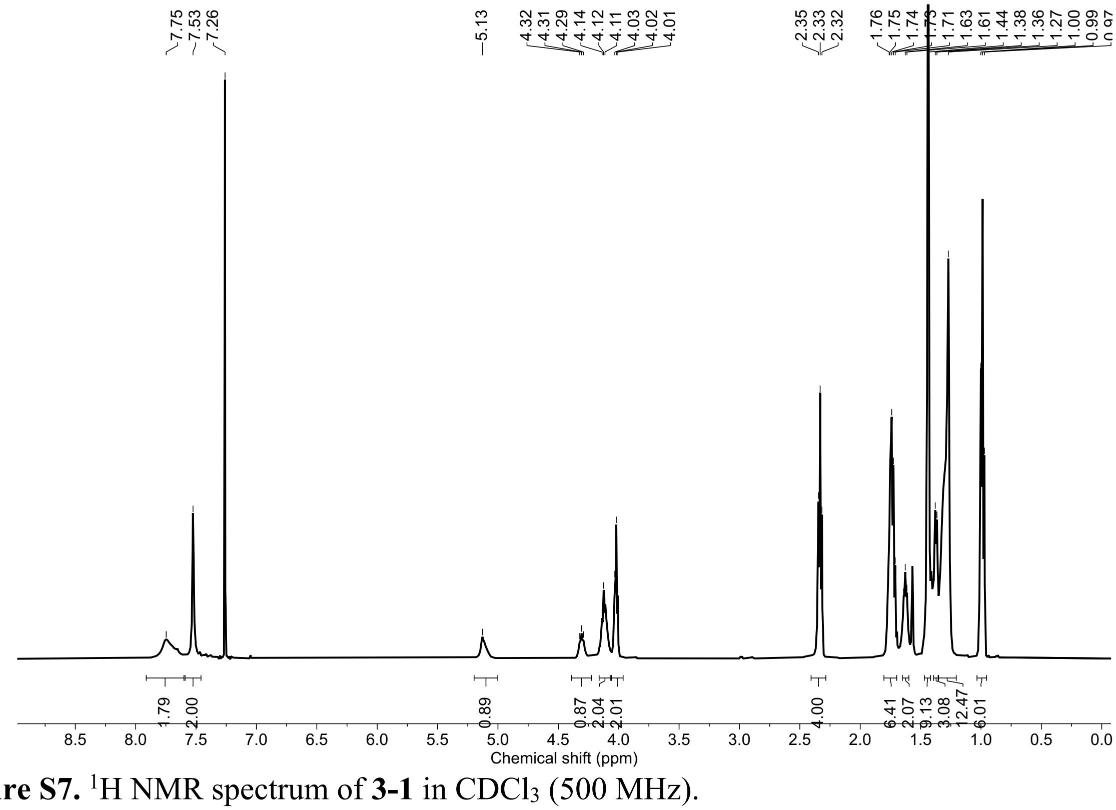

Figure S7. ^{1}H NMR spectrum of **3-1** in $CDCl_3$ (500 MHz).

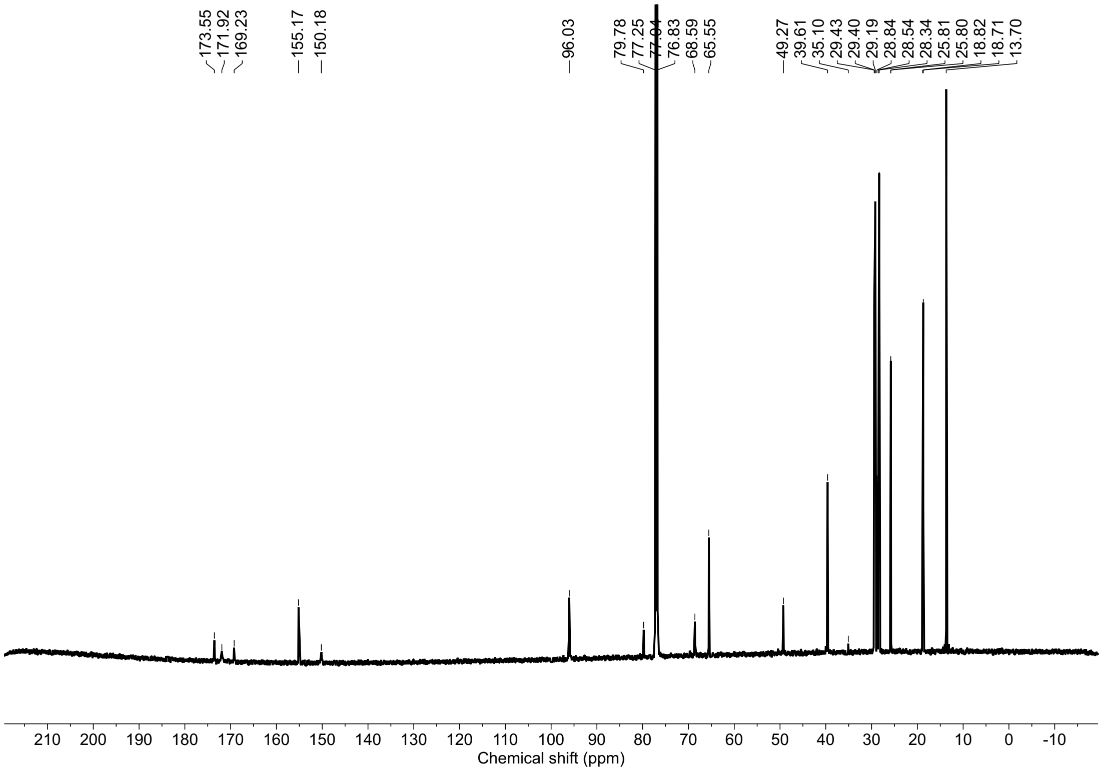

Figure S8. ^{13}C NMR spectrum of **3-1** in $CDCl_3$ (151 MHz).

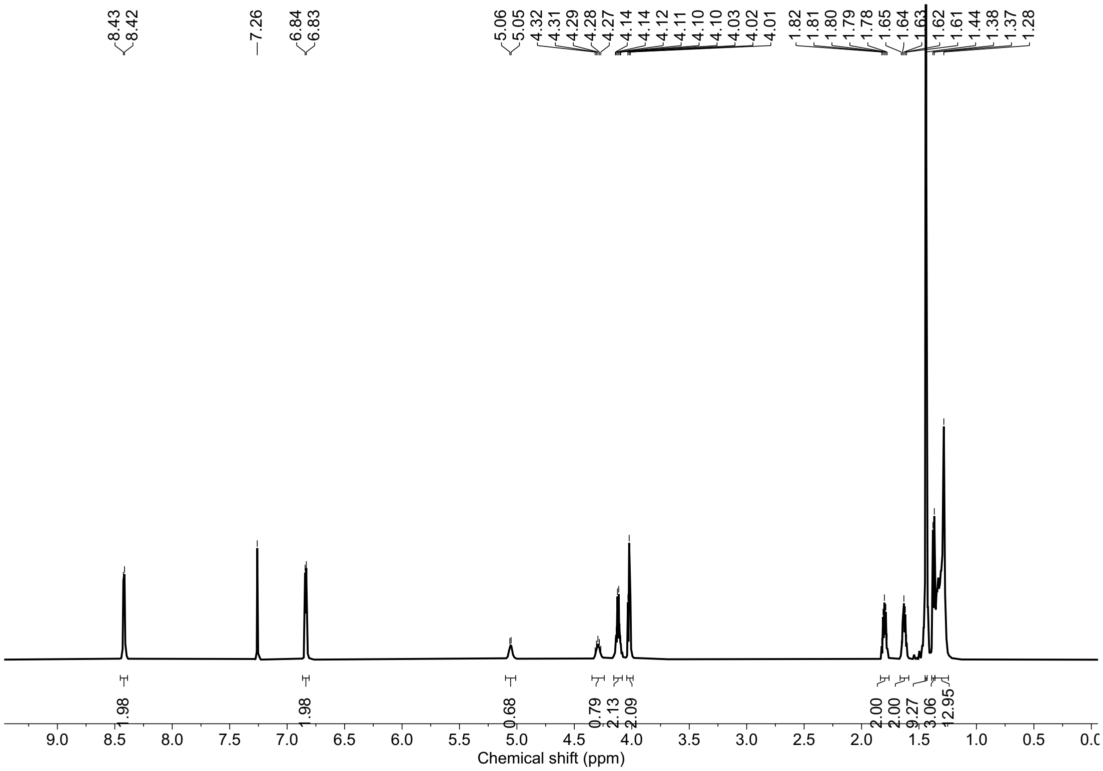

Figure S9. ^{1}H NMR spectrum of **3-2** in $CDCl_3$ (600 MHz).

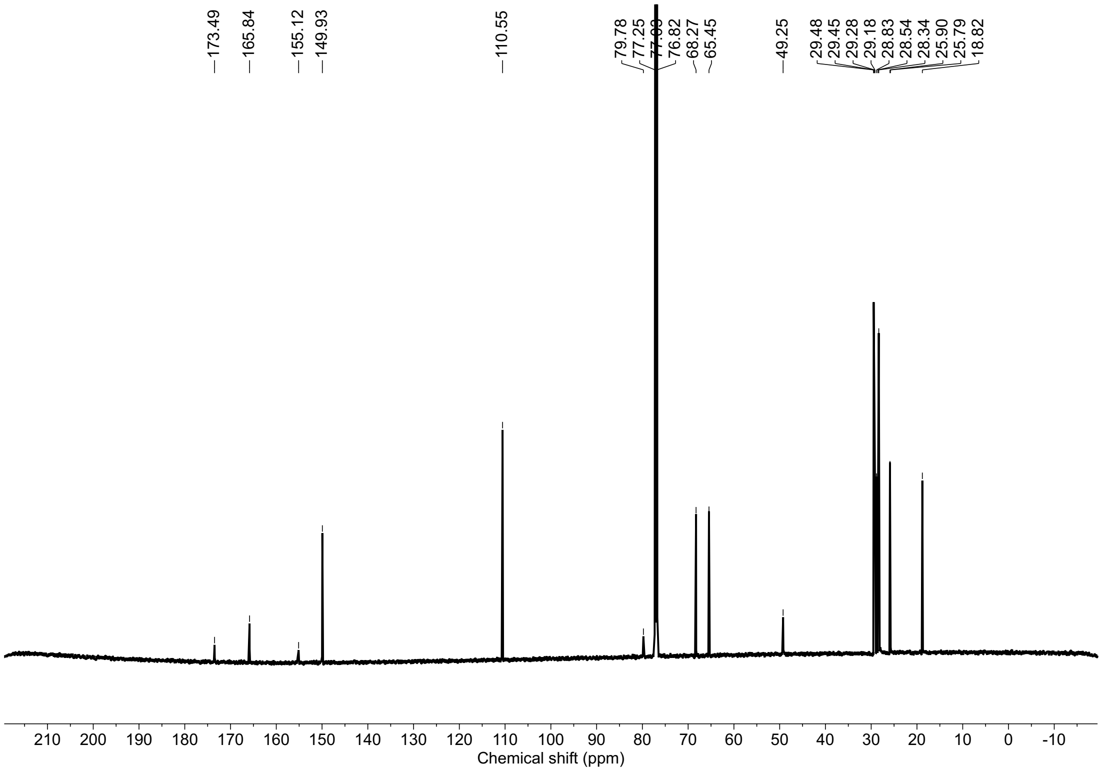

Figure S10. ^{13}C NMR spectrum of **3-2** in $CDCl_3$ (151 MHz).

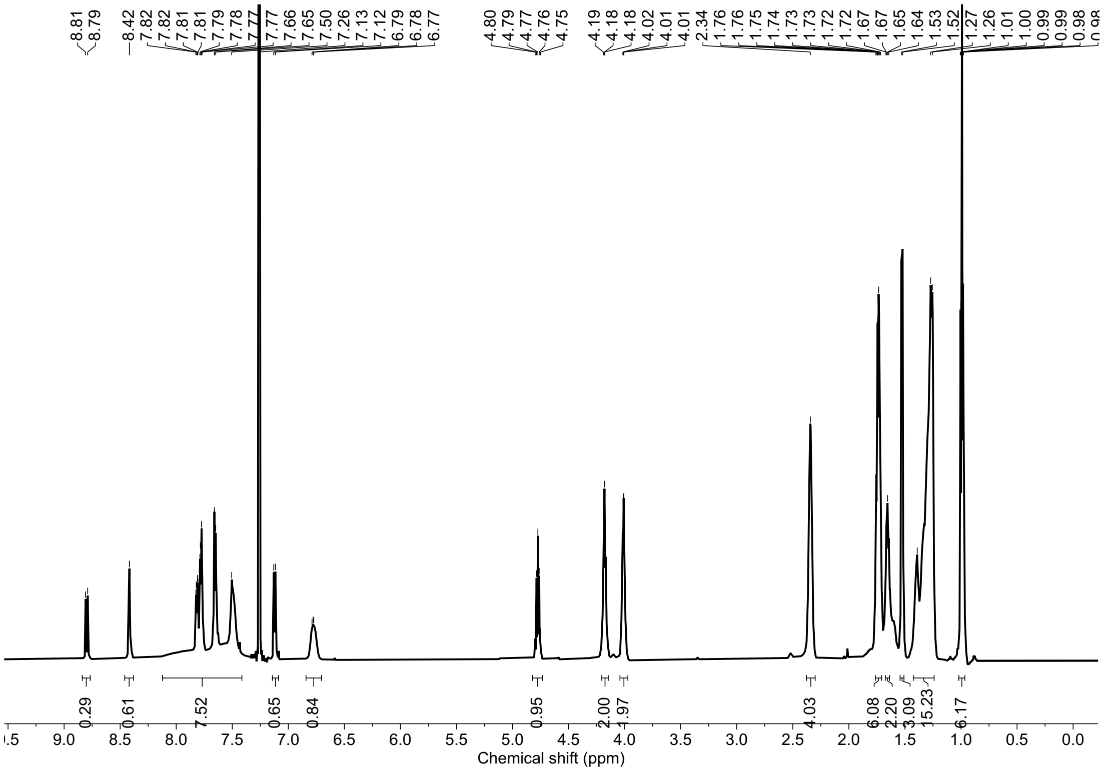

Figure S11. ^{1}H NMR spectrum of **4-1** in $CDCl_3$ (600 MHz).

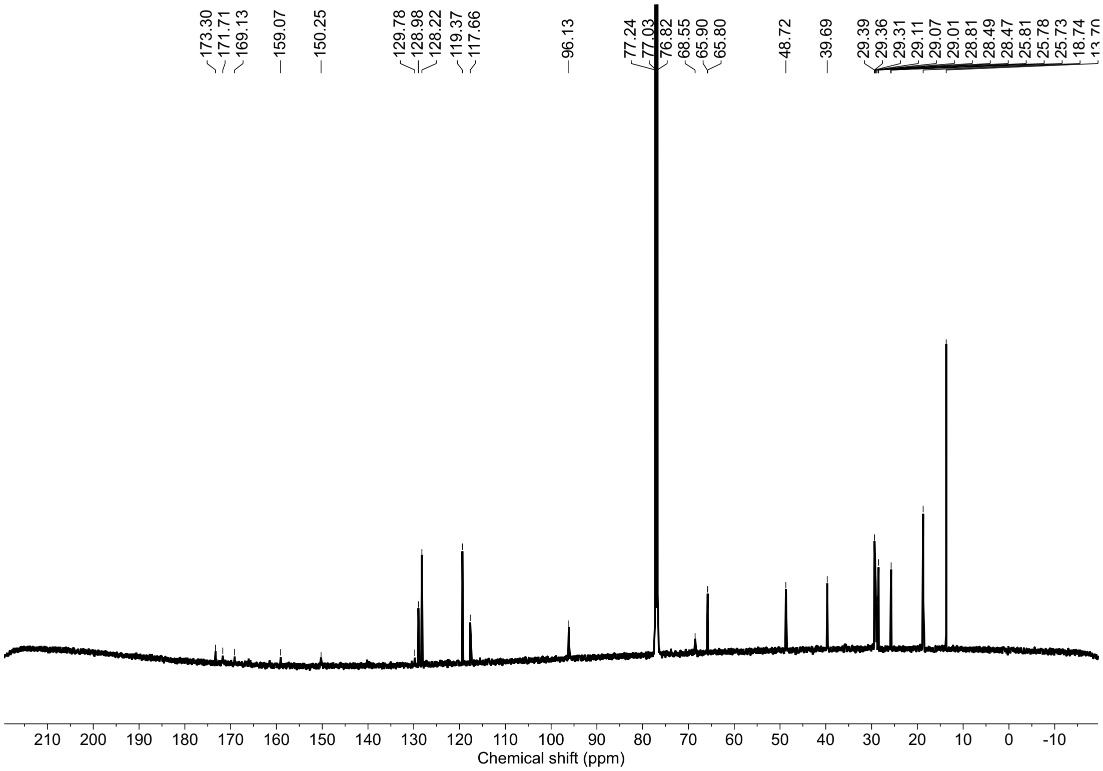

Figure S12. ^{13}C NMR spectrum of **4-1** in $CDCl_3$ (151 MHz).

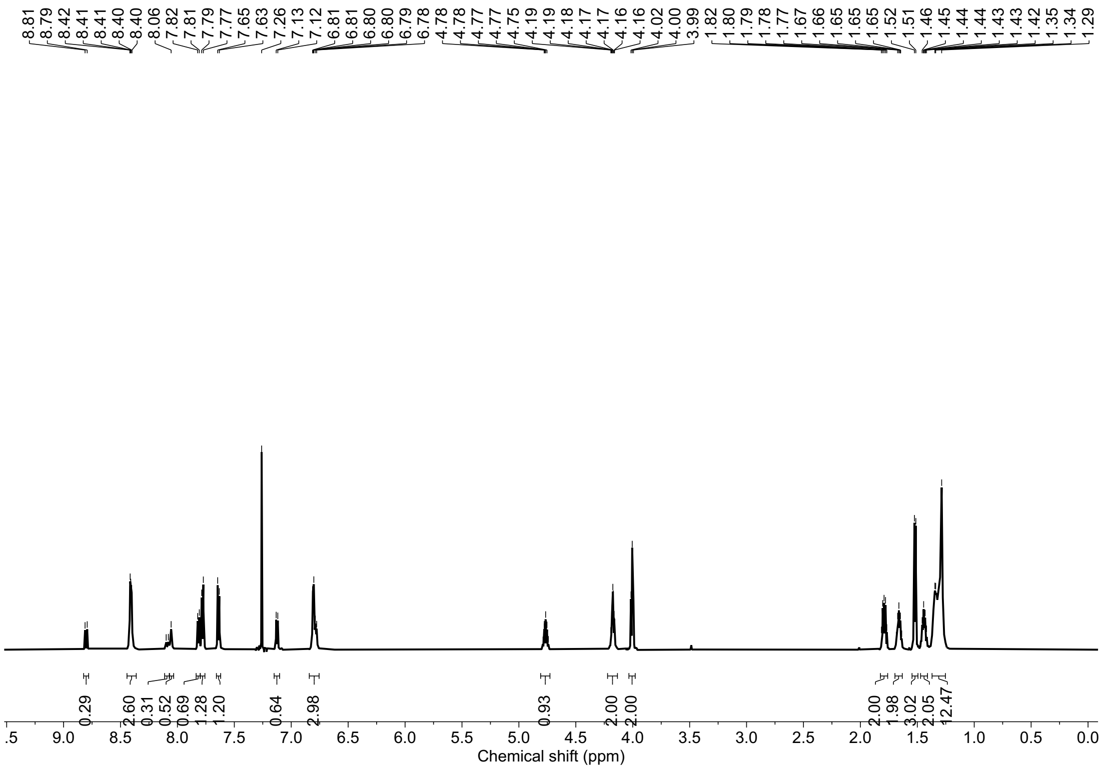

Figure S13. ^{1}H NMR spectrum of **4-2** in $CDCl_3$ (600 MHz).

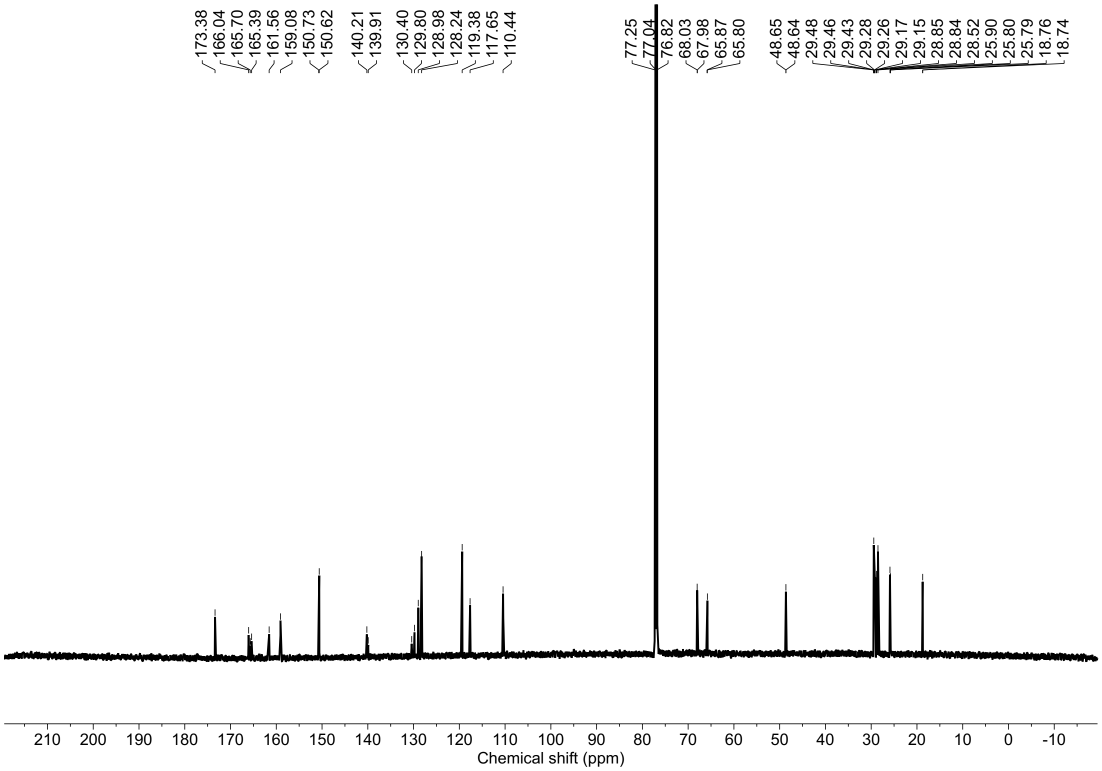

Figure S14. ^{13}C NMR spectrum of **4-2** in $CDCl_3$ (151 MHz).

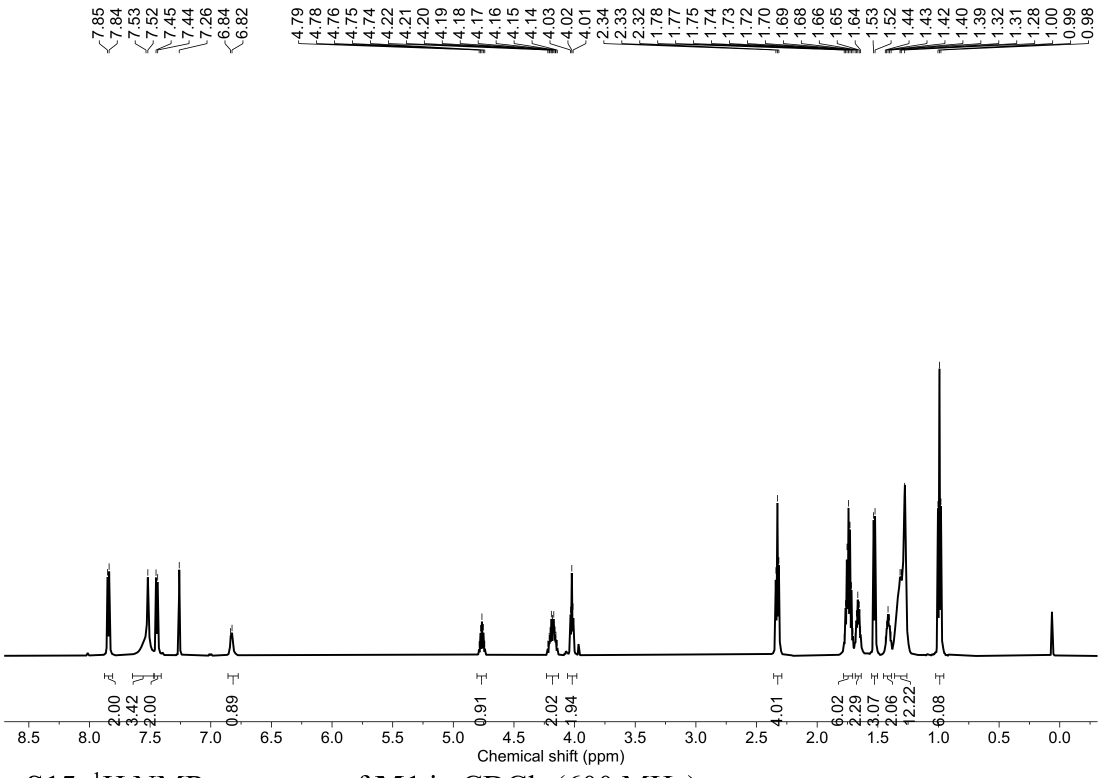

Figure S15. ^{1}H NMR spectrum of **M1** in $CDCl_3$ (600 MHz).

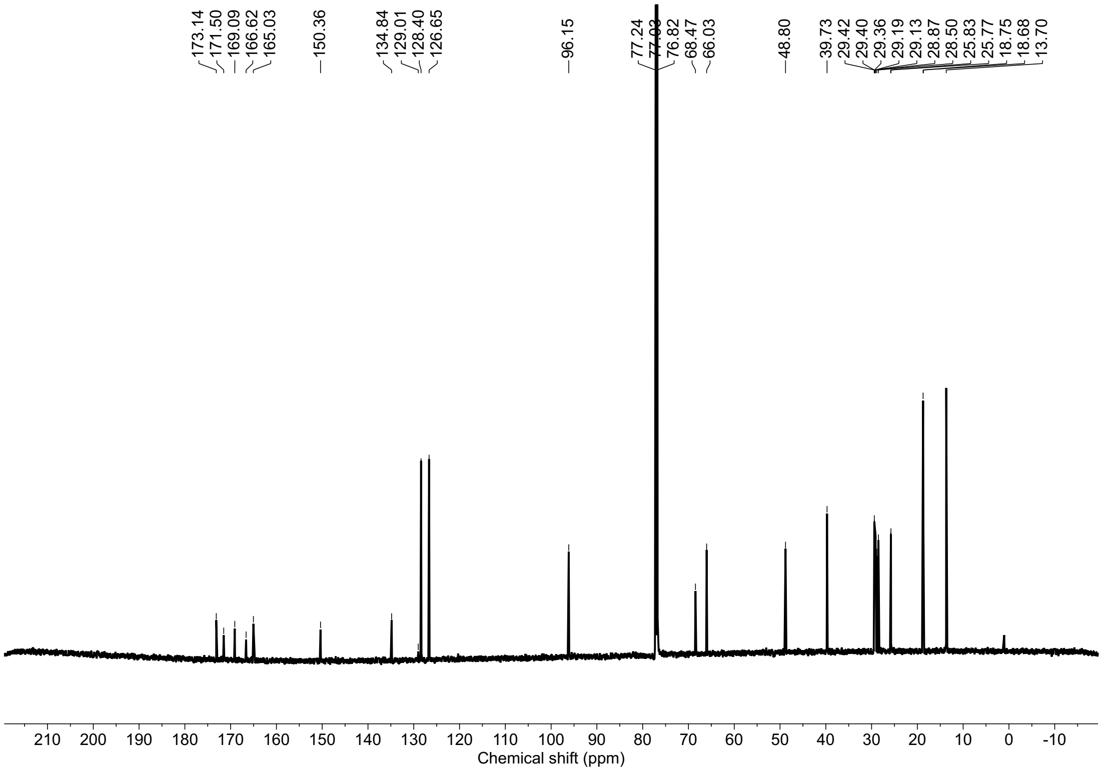

Figure S16. ^{13}C NMR spectrum of **M1** in $CDCl_3$ (151 MHz).

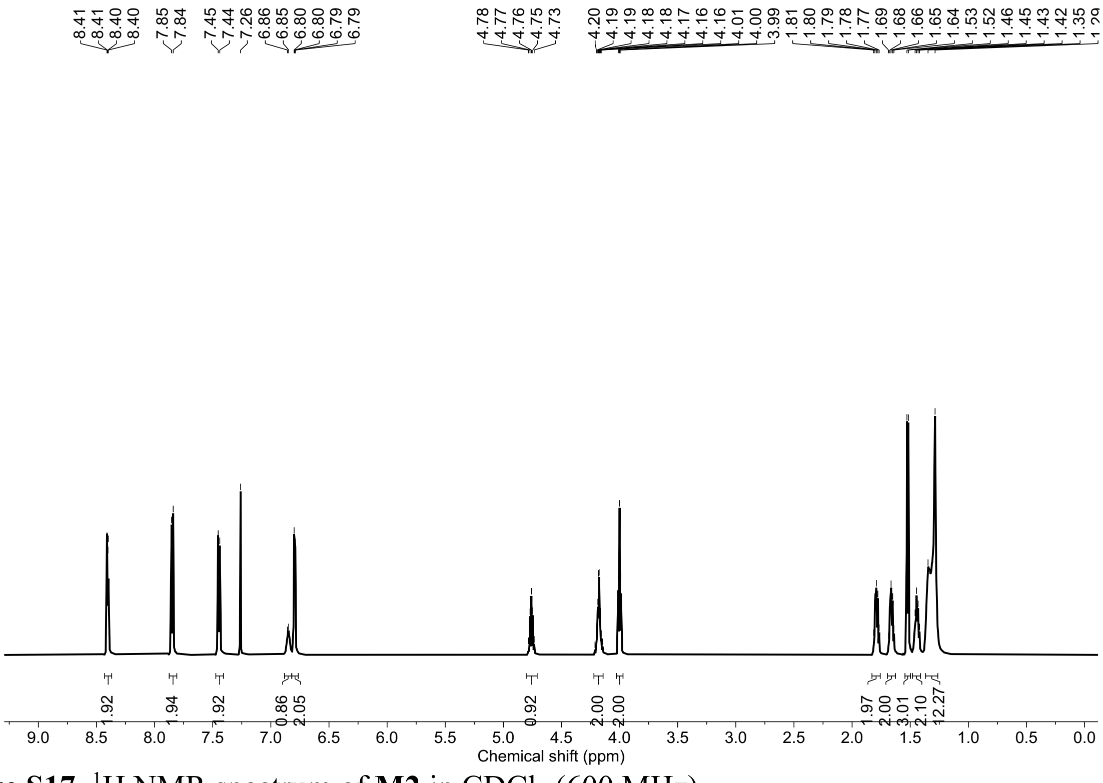

Figure S17. ^{1}H NMR spectrum of **M2** in $CDCl_3$ (600 MHz).

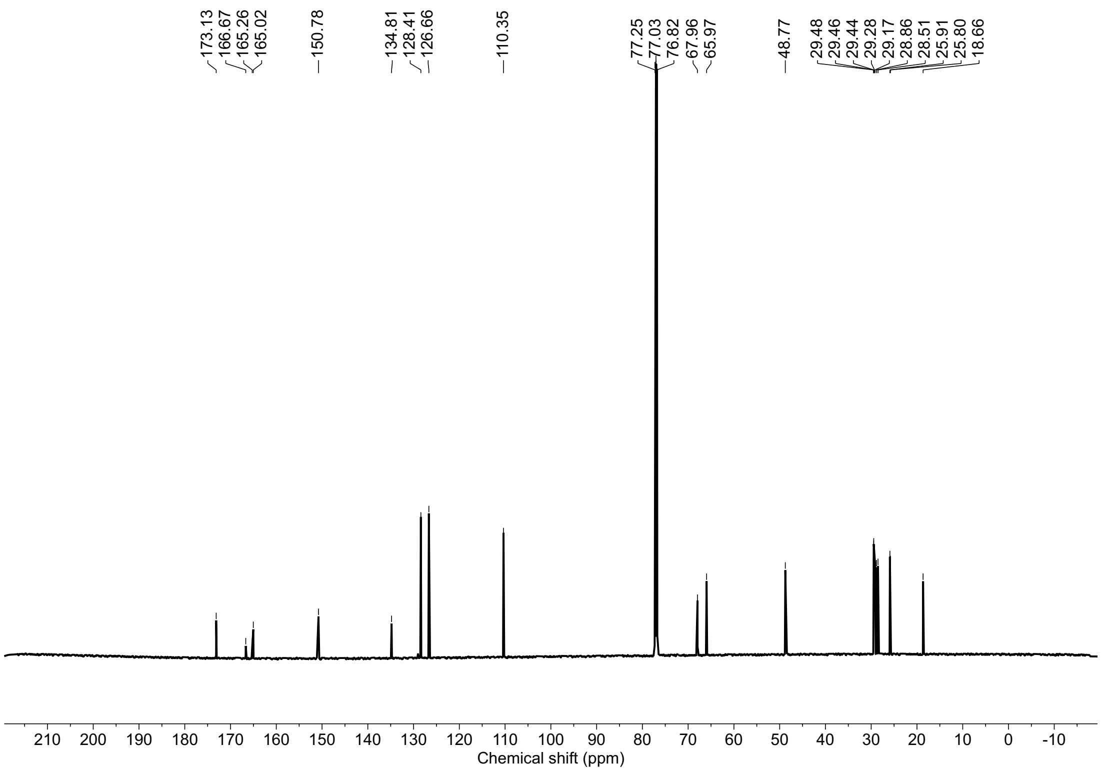

Figure S18. ^{13}C NMR spectrum of **M2** in $CDCl_3$ (151 MHz).

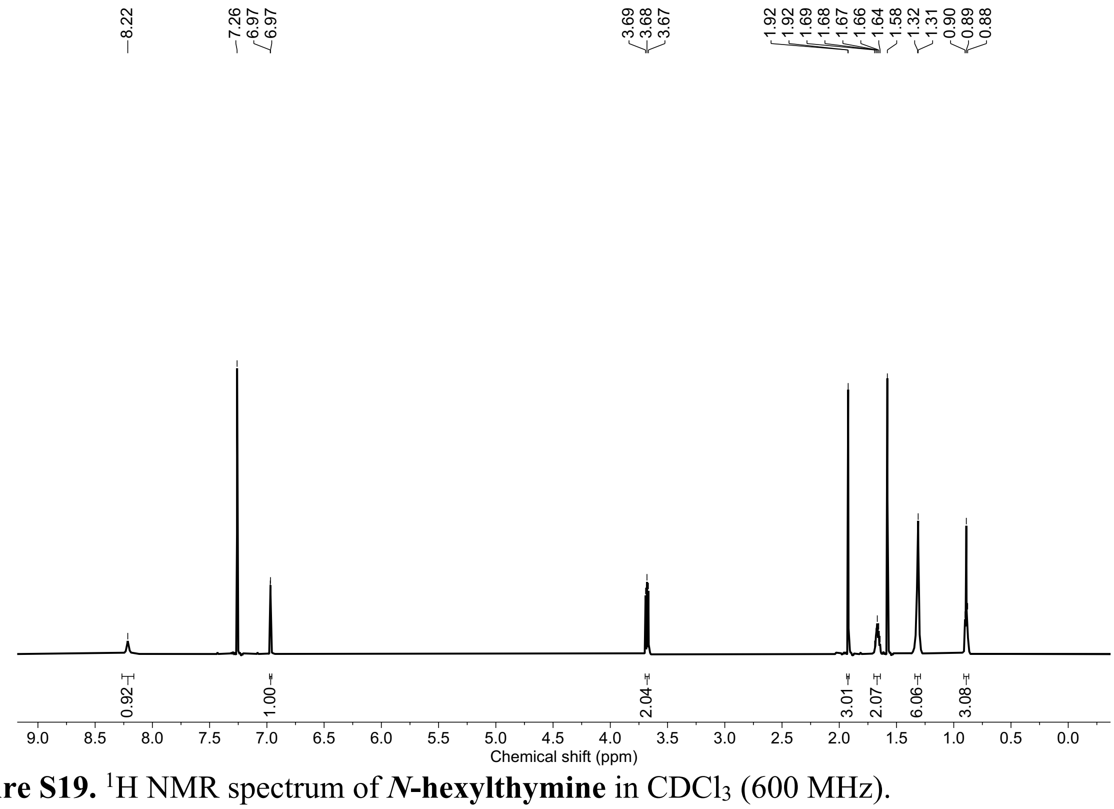

Figure S19. ^{1}H NMR spectrum of ***N*-hexylthymine** in $CDCl_3$ (600 MHz).

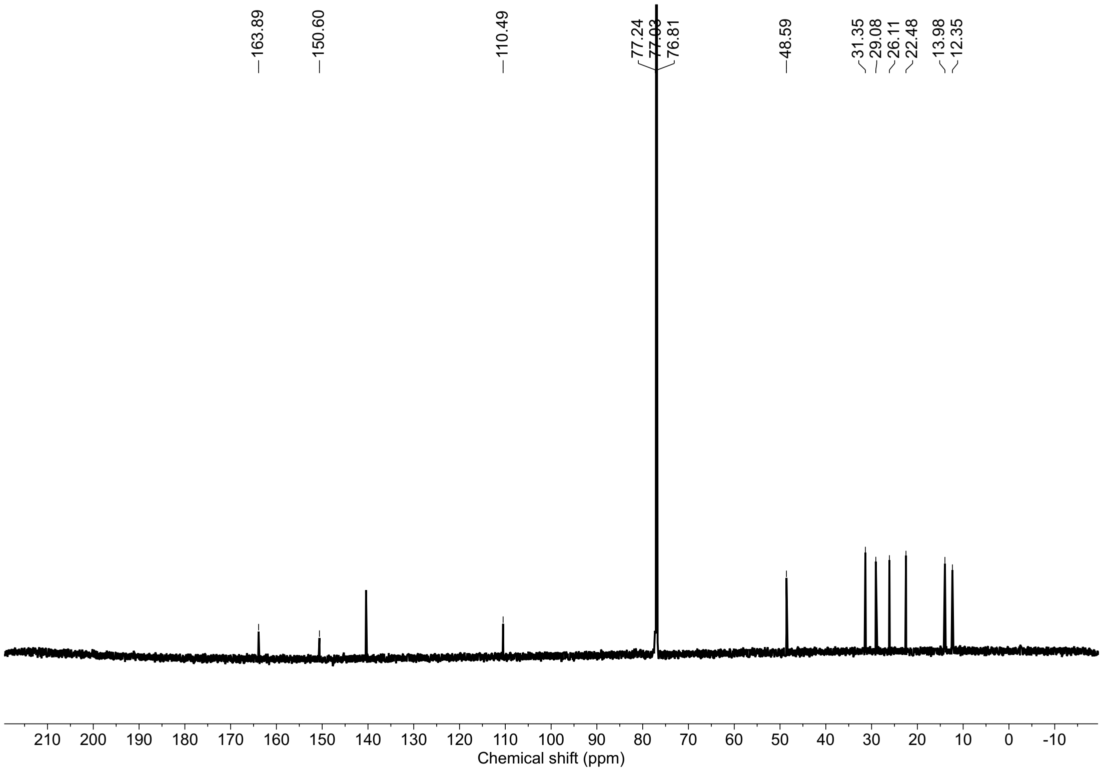

Figure S20. ^{13}C NMR spectrum of ***N*-hexylthymine** in $CDCl_3$ (151 MHz).

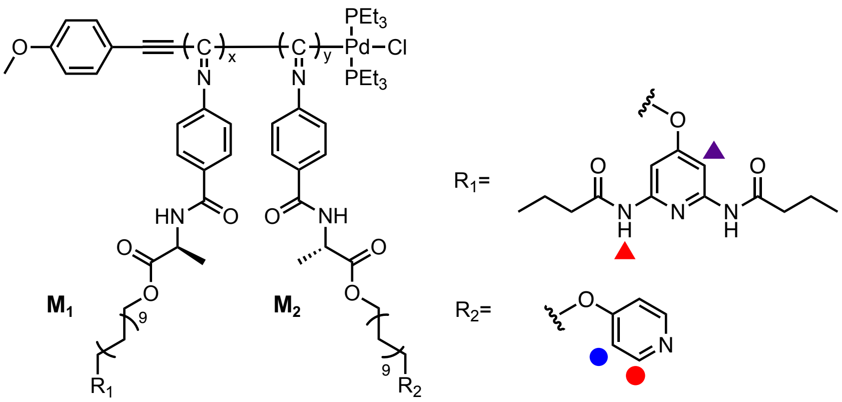

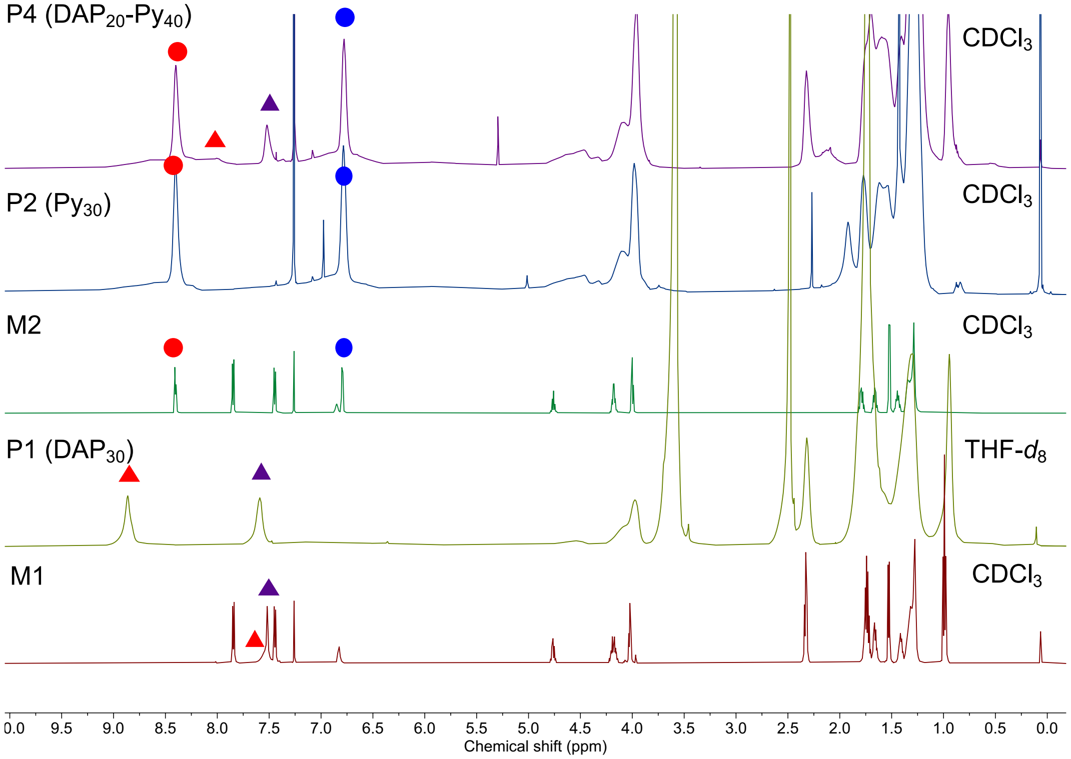

Figure S21. ^{1}H NMR spectra of **P1**, **P2**, **P4** and the comparison with **M1** and **M2** (600 MHz).

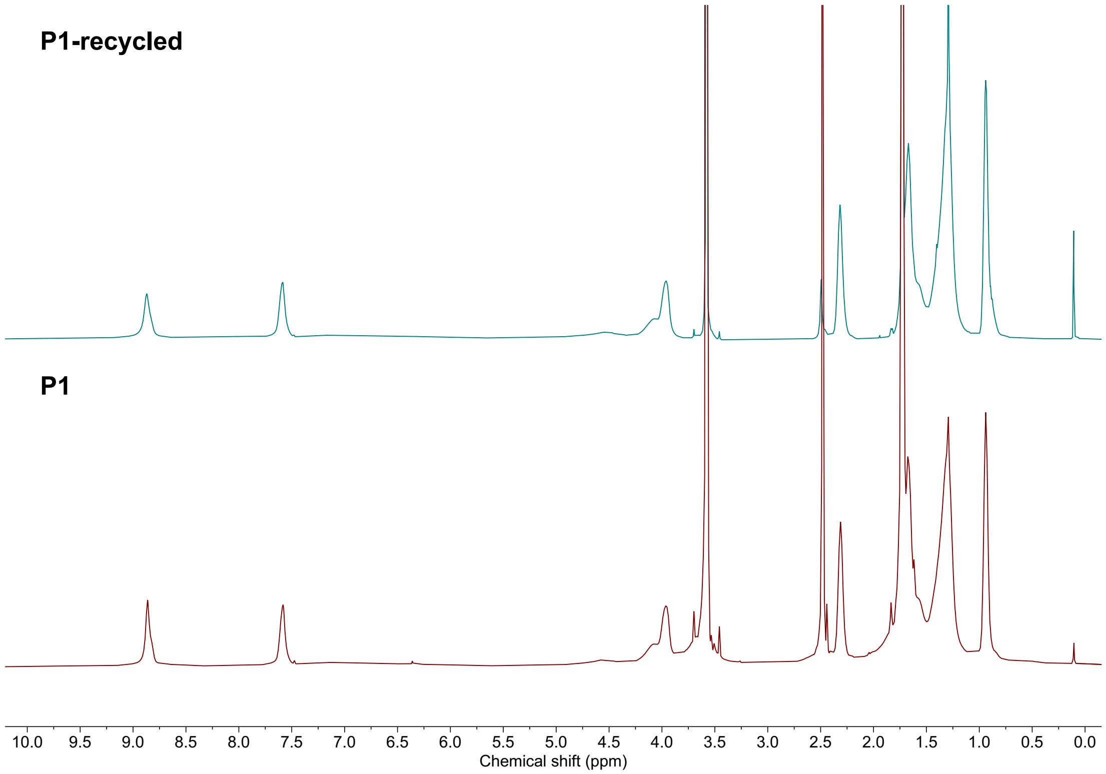

Figure S22. ^{1}H NMR spectra of original **P1** and recycled **P1** in THF-d_8 (600 MHz).

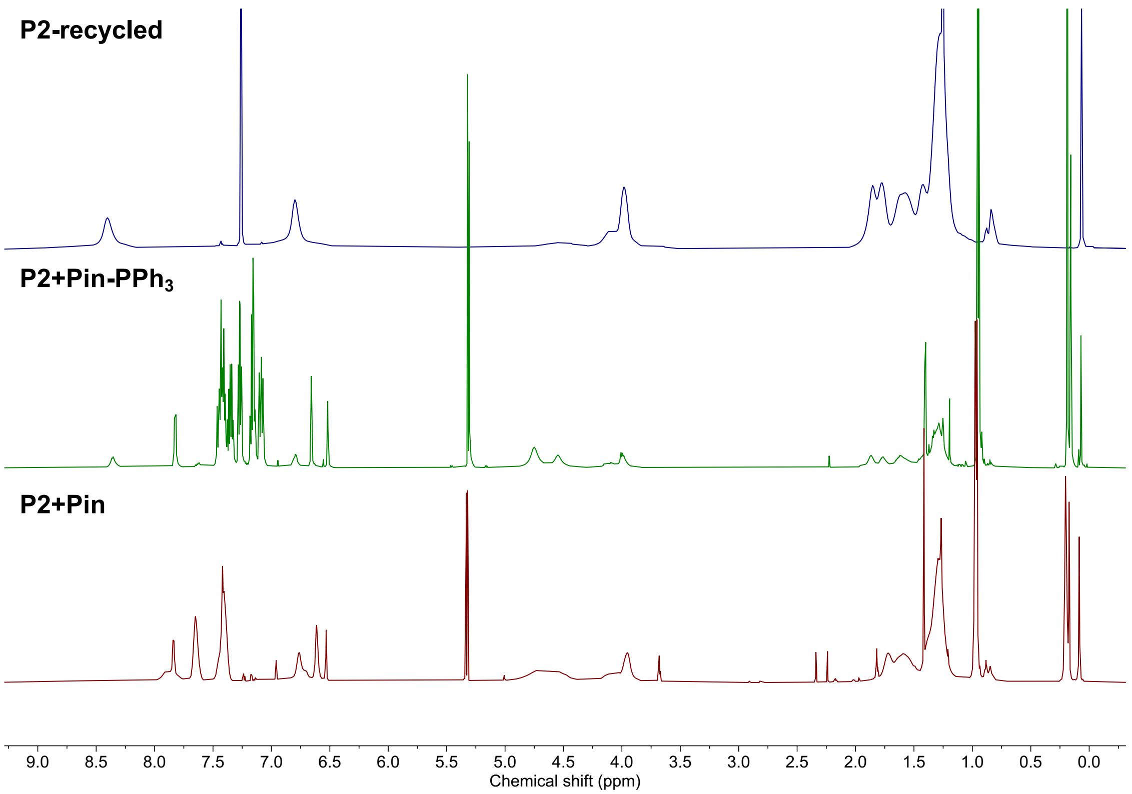

Figure S23. ^{1}H NMR spectra of **P2-Pin**, disassembled **P2+Pin-PPh$_3$** in CD_2Cl_2, and recycled **P2** in $CDCl_3$ (600 MHz).

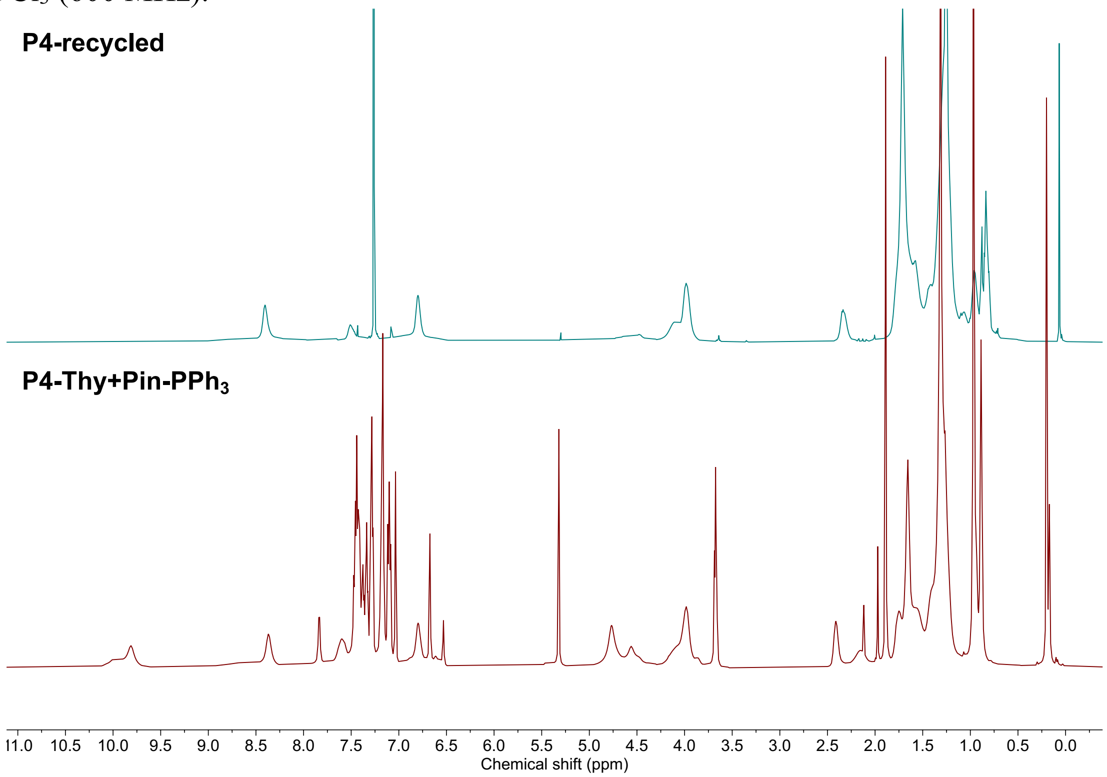

Figure S24. ^{1}H NMR spectra of disassembled **P4-Thy+Pin-PPh$_3$** in CD_2Cl_2 and recycled **P4** in $CDCl_3$ (600 MHz).

^{1}H NMR Titration experiments

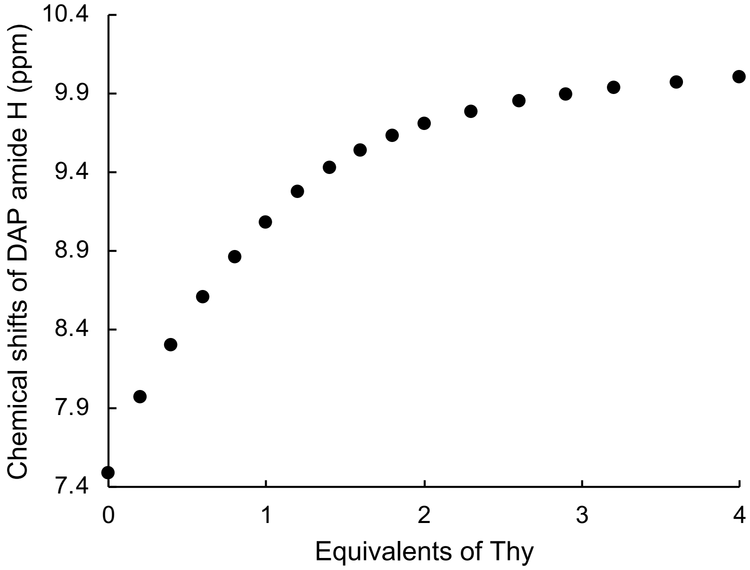

Figure S25. Titration curve of **M1** with *N*-hexylthymine in $CDCl_3$.

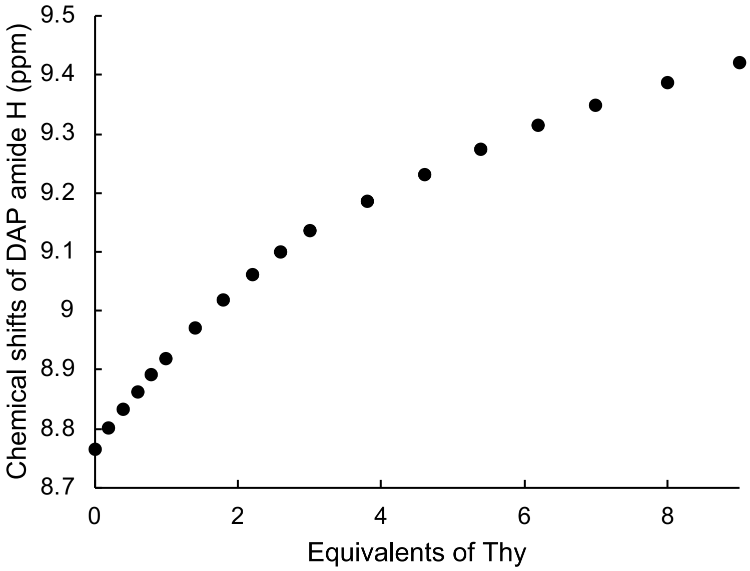

Figure S26. Titration curve of **M1** with *N*-hexylthymine in THF-d_8.

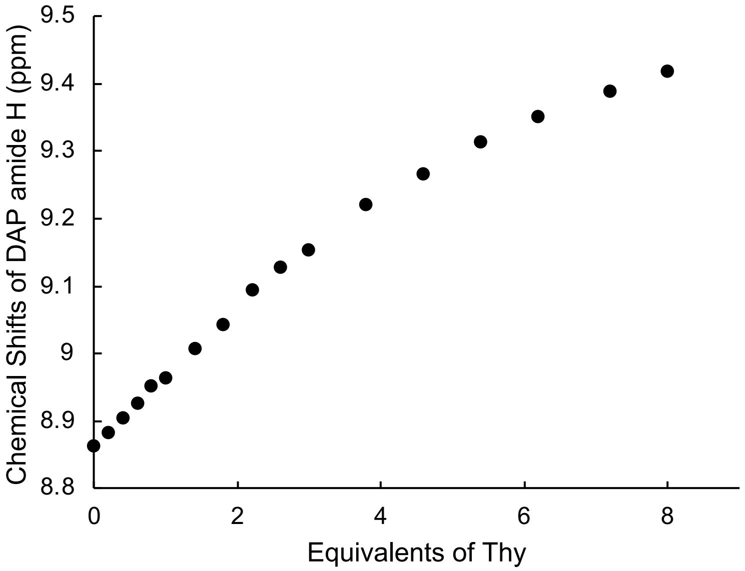

Figure S27. Titration curve of **P1** with *N*-hexylthymine in THF-d_8.

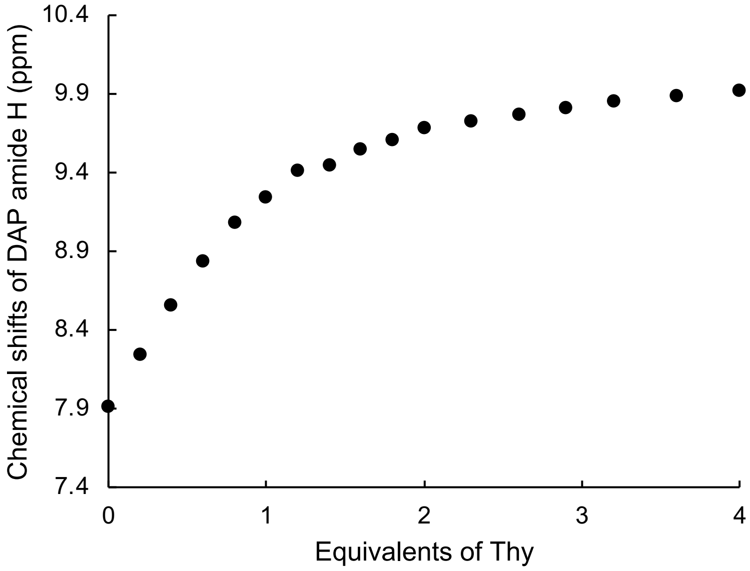

Figure S28. Titration curve of **P4** with *N*-hexylthymine in $CDCl_3$.

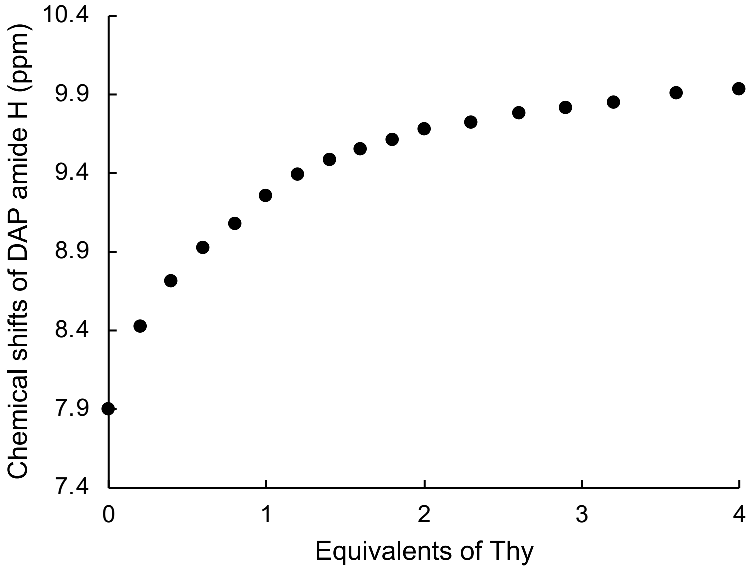

Figure S29. Titration curve of **P4-Pin** with *N*-hexylthymine in CD_2Cl_2.

CD spectra

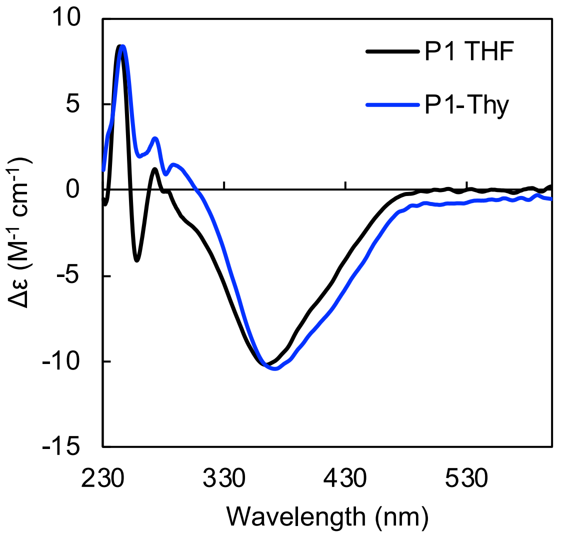

Figure S30. CD spectra of **P1** (black) in THF and **P1-Thy** (blue) in chloroform.

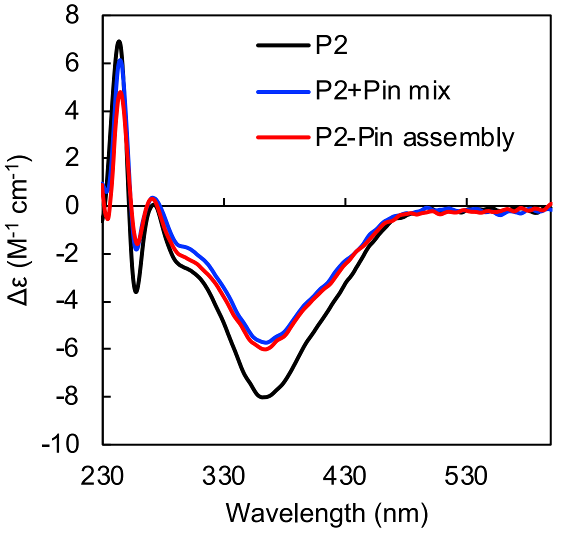

Figure S31. CD spectra of **P2** (black), **P2+Pin** physical mixture (blue) and **P2-Pin** assembly (red) in chloroform.

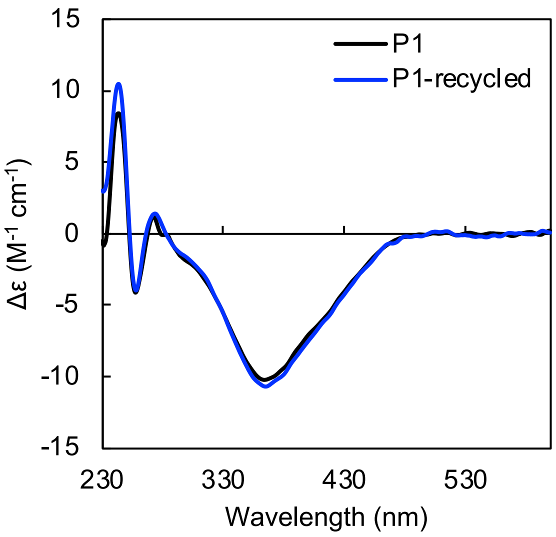

Figure S32. CD spectra of **P1** (black) and recycled **P1** (blue) in THF.

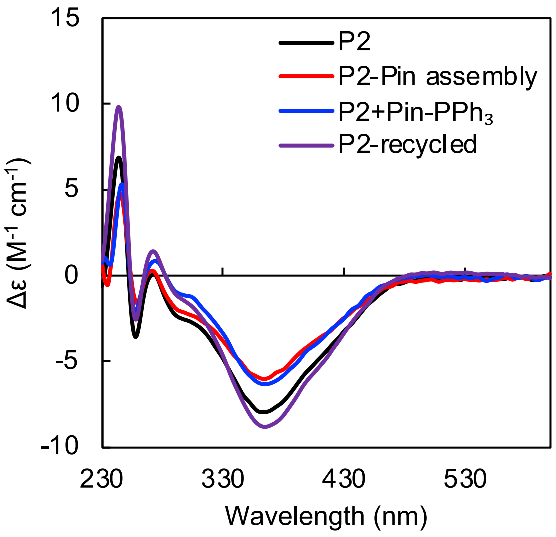

Figure S33. CD spectra of **P2** (black), **P2-Pin** assembly (red), dissembled **P2+Pin-PPh$_3$** (blue) and recycled **P2** (purple) in chloroform.

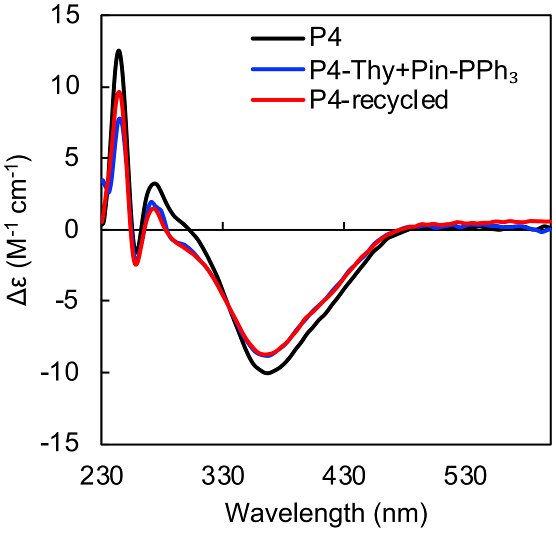

Figure S34. CD spectra of **P4** (black), dissembled **P4-Thy+Pin-PPh$_3$** (blue) and recycled **P4** (red) in chloroform.

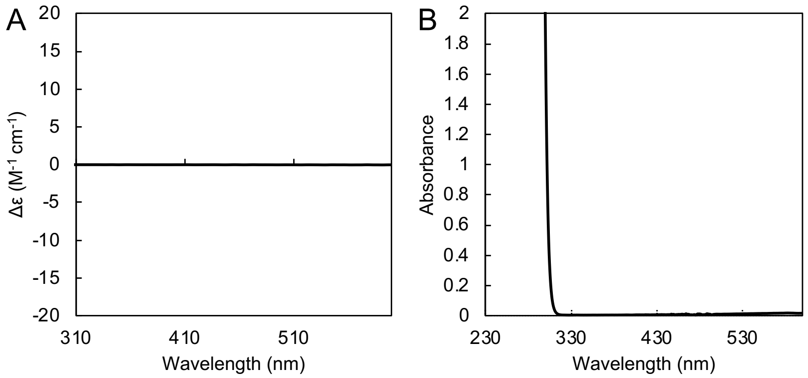

Figure S35. (A) CD and (B) UV spectra of *N*-hexylthymine (0.015 M) in chloroform.

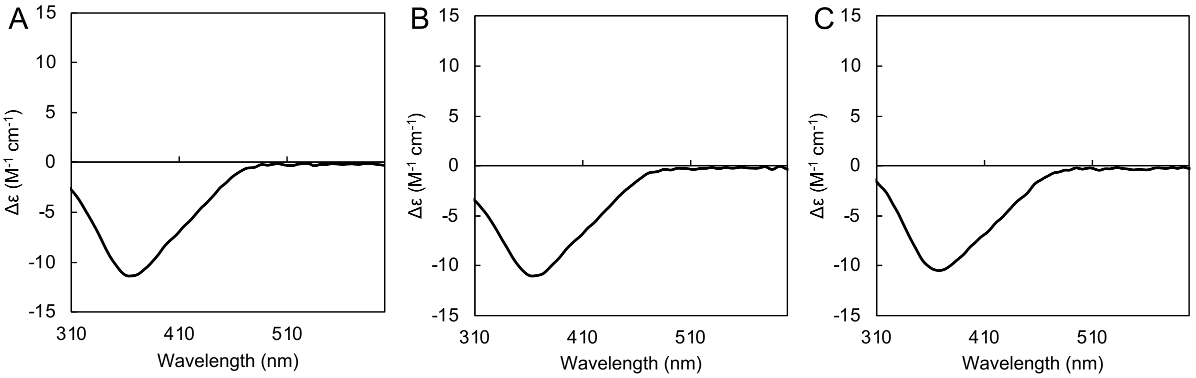

Figure S36. CD spectra of (A) **P1**, (B) **P4**, and (C) **P4-Pin** with a large excess of *N*-hexylthymine (0.014 M) in chloroform.

GPC traces

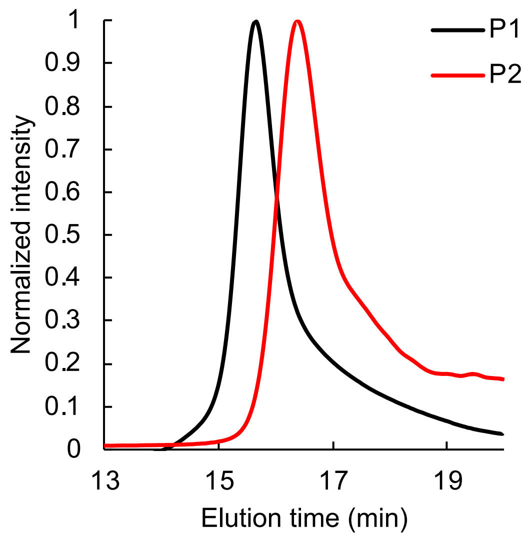

Figure S37. GPC traces of **P1** and **P2** using THF as the eluent.

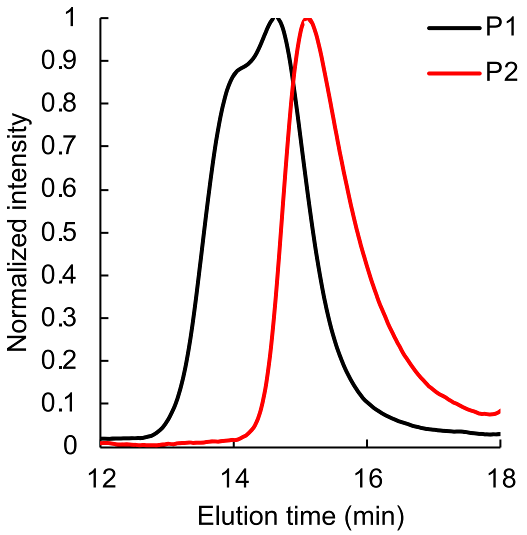

Figure S38. GPC traces of **P1** and **P2** using DMF with 0.03 M LiCl as the eluent.

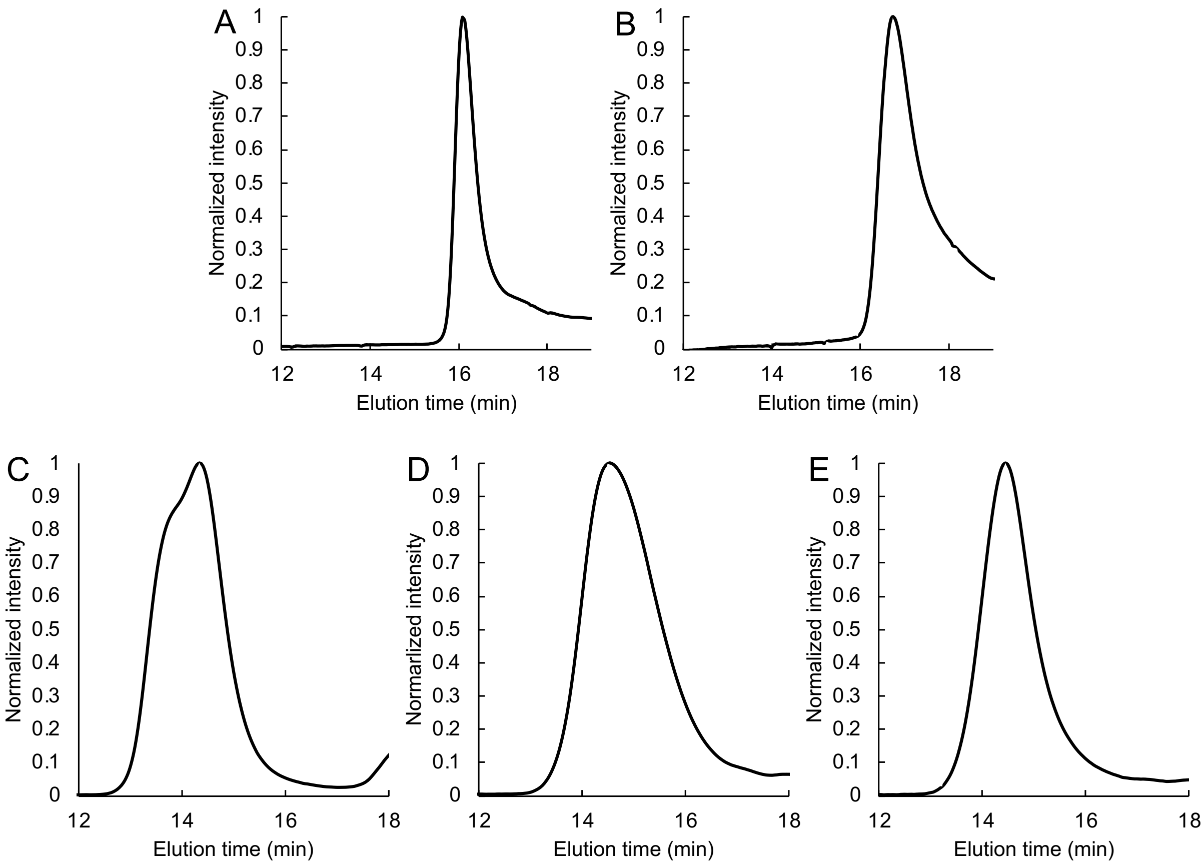

Figure S39. GPC traces of recycled (A) **P1** (M_n = 7.5 kDa, $Đ$ = 1.07) and (B) **P2** (M_n = 3.9 kDa, $Đ$ = 1.20) using THF as the eluent, (C) **P1** (M_n =18.0 kDa, $Đ$ = 1.74), (D) **P2** (M_n = 9.5 kDa, $Đ$ = 1.51), and (E) **P4** (M_n = 12.3 kDa, $Đ$ = 1.48) using DMF with 0.03 M LiCl as the eluent. The deviation of measured molecular weights and dispersities from the original polymers using DMF as the eluent was originated from a deteriorated column condition compared with the condition when the original polymers data in Table 1 was measured. The original **P2** sample measured in the deteriorated condition showed M_n = 9.4 kDa, $Đ$ = 1.60.

References

(1) Stubbs, L. P.; Weck, M., Towards a Universal Polymer Backbone: Design and Synthesis of Polymeric Scaffolds Containing Terminal Hydrogen-Bonding Recognition Motifs at Each Repeating Unit. *Chem. Eur. J.* **2003,** *9*, 992-999.

(2) Kajitani, T.; Okoshi, K.; Sakurai, S.-i.; Kumaki, J.; Yashima, E., Helix-Sense Controlled Polymerization of a Single Phenyl Isocyanide Enantiomer Leading to Diastereomeric Helical Polyisocyanides with Opposite Helix-Sense and Cholesteric Liquid Crystals with Opposite Twist-Sense. *J. Am. Chem. Soc.* **2006,** *128*, 708-709.

(3) Deng, R.; Wang, C.; Milton, M.; Tang, D.; Hollingsworth, A. D.; Weck, M., Side-chain functionalized supramolecular helical brush copolymers. *Polym. Chem.* **2021,** *12*, 4916-4923.
